# Cross-Reactivity to Mutated Viral Immune Targets Can Influence CD8^+^ T Cell Functionality: An Alternative Viral Adaptation Strategy

**DOI:** 10.3389/fimmu.2021.746986

**Published:** 2021-10-26

**Authors:** Jennifer Currenti, Becker M.P. Law, Kai Qin, Mina John, Mark A. Pilkinton, Anju Bansal, Shay Leary, Ramesh Ram, Abha Chopra, Rama Gangula, Ling Yue, Christian Warren, Louise Barnett, Eric Alves, Wyatt J. McDonnell, Anuradha Sooda, Sonya L. Heath, Simon Mallal, Paul Goepfert, Spyros A. Kalams, Silvana Gaudieri

**Affiliations:** ^1^School of Human Sciences, University of Western Australia, Crawley, WA, Australia; ^2^Department of Medicine, University of Alabama at Birmingham, Birmingham, AL, United States; ^3^Institute for Immunology and Infectious Diseases, Murdoch University, Murdoch, WA, Australia; ^4^Department of Clinical Immunology, Royal Perth Hospital, Perth, WA, Australia; ^5^Division of Infectious Diseases, Department of Medicine, Vanderbilt University Medical Center, Nashville, TN, United States; ^6^Emory Vaccine Center at Yerkes National Primate Research Center, Emory University, Atlanta, GA, United States; ^7^Department of Pathology and Laboratory Medicine, Emory University, Atlanta, GA, United States

**Keywords:** HIV, adaptation, host-viral interactions, T cell receptor, transcriptome

## Abstract

Loss of T cell immunogenicity due to mutations in virally encoded epitopes is a well-described adaptation strategy to limit host anti-viral immunity. Another described, but less understood, adaptation strategy involves the selection of mutations within epitopes that retain immune recognition, suggesting a benefit for the virus despite continued immune pressure (termed non-classical adaptation). To understand this adaptation strategy, we utilized a single cell transcriptomic approach to identify features of the HIV-specific CD8^+^ T cell responses targeting non-adapted (NAE) and adapted (AE) forms of epitopes containing a non-classical adaptation. T cell receptor (TCR) repertoire and transcriptome were obtained from antigen-specific CD8^+^ T cells of chronic (n=7) and acute (n=4) HIV-infected subjects identified by either HLA class I tetramers or upregulation of activation markers following peptide stimulation. CD8^+^ T cells were predominantly dual tetramer^+^, confirming a large proportion of cross-reactive TCR clonotypes capable of recognizing the NAE and AE form. However, single-reactive CD8^+^ T cells were identified in acute HIV-infected subjects only, providing the potential for the selection of T cell clones over time. The transcriptomic profile of CD8^+^ T cells was dependent on the autologous virus: subjects whose virus encoded the NAE form of the epitope (and who transitioned to the AE form at a later timepoint) exhibited an ‘effective’ immune response, as indicated by expression of transcripts associated with polyfunctionality, cytotoxicity and apoptosis (largely driven by the genes GZMB, IFNɣ, CCL3, CCL4 and CCL5). These data suggest that viral adaptation at a single amino acid residue can provide an alternative strategy for viral survival by modulating the transcriptome of CD8^+^ T cells and potentially selecting for less effective T cell clones from the acute to chronic phase.

## Introduction

The rapid mutation rate of human immunodeficiency virus (HIV) remains a central barrier to an effective vaccine, as selection of mutations within the HIV genome by effective antigen-specific CD8^+^ T cell immune responses (termed adaptations) causes failure of otherwise potent natural or vaccine-induced immunity. This form of immune selection pressure is one of the main drivers shaping viral quasispecies during the course of natural infection, highlighting the important role of adaptation in viral pathogenesis (1–3). Understanding HIV adaptation to T cell responses is critical to identifying correlates of immune efficacy against HIV and will aid in the rational design of potential vaccine candidates for either preventative or therapeutic strategies. However, the mechanisms underpinning HIV adaptation to T cell immunity are not completely understood.

HIV adaptations are positively selected in infected individuals and are also apparent at the population level as human leucocyte antigen (HLA)-specific HIV polymorphisms (4) as the host’s HLA allelic repertoire determine the pathogen-derived peptides (epitopes) that are presented to T cells. Epitopes containing these HLA-associated polymorphisms have been termed adapted epitopes (AE), and those epitopes lacking any evidence of HLA-associated changes non-adapted epitopes (NAE). Prior studies have shown that many viral adaptations, termed here as ‘classical’ adaptation, can completely abrogate T cell recognition *via* disrupting the HLA-peptide-T cell receptor (TCR) complex or antigen processing [(5, 6) and reviewed in (7, 8)]. Our previous large-scale analysis of epitope-specific CD8^+^ T cell responses confirmed the classical form of adaptation for many variable sites across the HIV proteome, with the adaptations largely occurring at anchor residues known to influence the binding efficacy of the HLA-peptide complex (9). However, epitopes containing adaptations at non-anchor residue positions continue to be targeted by CD8^+^ T cells, suggesting alternative non-classical adaptation strategies exploited by HIV (10), and indeed by other mutable pathogens such as hepatitis C virus (11) and others (12, 13), to subvert the host’s immune response.

In some cases of non-classical HIV adaptation, CD8^+^ T cells exhibited greater functional avidity, as measured by interferon gamma (IFNγ) release, to the AE form of the epitope than the NAE form (14, 15). On the contrary, using a similar IFNγ release assay, other studies have demonstrated that in both acute and chronic HIV infection, NAEs harbor greater functional avidity (15, 16). Although functional avidity has been shown to be a powerful predictor for T cell polyfunctionality due to IFNɣ release, such discrepancies among different studies highlight the necessity for a more direct functional assessment of T cells that target the NAE and AE forms (17). Another example of an adaptation mechanism is the formation of ‘neo-epitopes’ due to the adaptation exposing new targets to the immune system, often in the more variable protein Nef (10, 14). The development of ‘neo-epitopes’ could cause a shift in immunodominance, potentially compromising otherwise favorable responses against more conserved structural proteins like Gag and Pol. Importantly, if such epitopes containing these non-classical adaptations (and/or neo-epitopes) are included as a vaccine immunogen, the induced immune response may paradoxically lead to greater viral survival, as suggested by our recent study showing continued recognition of the AE form of HIV T cell epitopes likely increases viral *trans*-infection of CD4^+^ T cells (15). Furthermore, others have shown differences in the level of anti-HIV protection exerted by epitope-specific T cell responses, suggesting the active exclusion of select T cell epitopes from candidate HIV vaccines (18).

**Figure 1:**
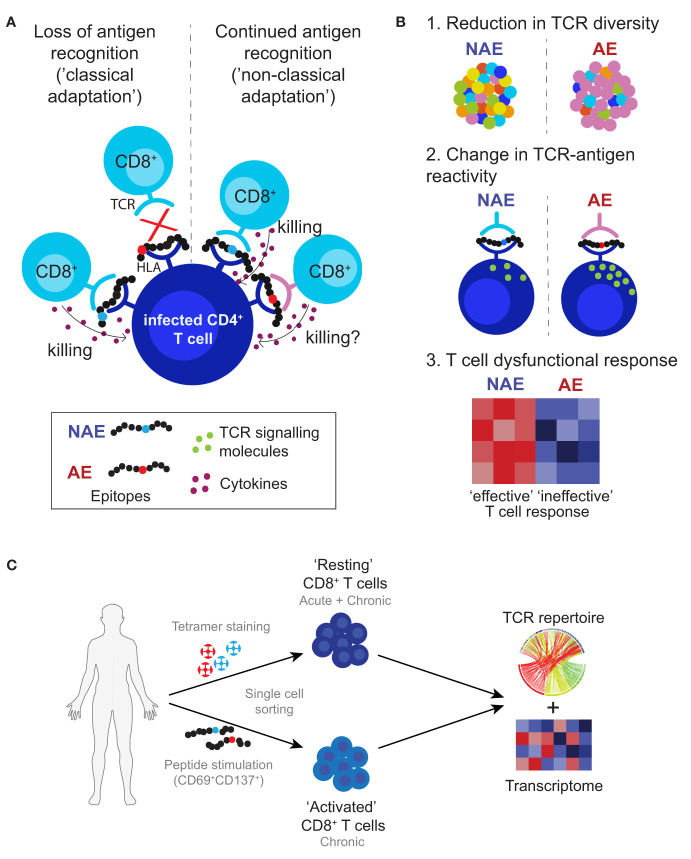
Proposed viral adaptation strategies against anti-HIV T cell immune responses. **(A)** Schematic depicts classical and non-classical adaptation based on recognition of the HLA-peptide complex by the TCR. **(B)** Lists the three main proposed mechanisms of non-classical adaptation tested in this study: 1. reduction in TCR diversity or activation of new TCR clonotypes; 2. differences in TCR-peptide-HLA antigen specific reactivity that affect T cell signalling; and 3. alterations in the interaction between the TCR and HLA-peptide complex leading to ineffective T cell responses. **(C)** Approach used in this study to identify and characterize antigen-specific CD8^+^ T cells. Cells were single cell sorted using HLA class I tetramers for the NAE- and AE-form of an epitope (assigned as ‘resting’ CD8^+^ T cells) and/or based on CD69 and CD137 expression after peptide stimulation with either the NAE or AE form of an epitope (assigned as ‘activated’ CD8^+^ T cells). All cells then underwent TCR sequencing and for select epitopes RNA sequencing to assess TCR repertoire and transcriptome profiles, respectively.

The non-classical form of adaptations often occur at sites within the epitope that may affect TCR recognition of the HLA-peptide complex and alter T cell functionality or TCR repertoire diversity; factors associated with control of pathogens that cause chronic infection (19–21). Our earlier study examining T cell responses against HIV T cell epitopes that exhibit these non-classical adaptations (14) was not able to identify differences in TCR repertoire, in part, due to the low-resolution nature of the bulk analysis of the antigen-specific T cells. Here, we utilized single-cell RNA sequencing to compare the TCR α/β sequences and gene expression (transcriptome) profiles of antigen-specific CD8^+^ T cells to the NAE and/or AE form of well-characterized CD8^+^ T cell epitopes. This approach allowed the direct link of T cell phenotype and specificity with T cell transcriptome data to elucidate alternative viral adaptation strategies that may encompass differences in TCR diversity and functional plasticity of antigen-specific CD8^+^ T cells (see **Figure 1** for adaptation strategies tested in this study). Overall, single cell TCR analyses demonstrated mainly cross-reactive memory CD8^+^ T cells capable of recognizing both NAE and AE forms of the T cell epitopes tested during acute and chronic HIV infection. However, the transcriptomic profile of these cross-reactive memory CD8^+^ T cells in both acute and chronic HIV-infected subjects was largely influenced by the adaptation status of the autologous form of the epitope within an individual, with the autologous NAE form (with the propensity to adapt) associated with greater CD8^+^ T cell effector function potential.

## Results

To examine the mechanisms of non-classical adaptation as indicated in **Figure 1**, CD8^+^ T cell epitopes within HIV were selected based on prior data from our group that showed adaptation did not reduce T cell recognition of the epitope and subsequent IFNɣ production (14, 15). The following HIV T cell epitopes in Nef were examined: TL10, KY11, RF10 and TY8. Overall, seven chronic and four acute HIV-infected subjects were assessed for epitope-specific CD8^+^ T cell responses to one or more of the epitopes listed above (**Table 1**; see *Materials and Methods* for details). Given the differences in HLA alleles and the breadth of CD8^+^ T cell responses between subjects, not all subjects could be tested for the same epitope and only the epitope RF10 could be tested in both the chronic and acute phases of HIV infection.

**Table 1:** Clinical, demographic and virological data of study subjects^*^.

Subject	Sex	Ethnicity	Age at sort (y)	Infection status	Estimated time of infection prior to sort	Epitope (protein and location; HLA restriction)	Autologous virus at sort(%)^#^	Autologous virus at last time point (years in sort)^$^	Viral load(log_10_copies/ml)	CD4^+^ T cell count (cells/mm^3^ )
A1	M	AA	25	Acute	46d	TY8 (Nef 128-135; B*3501)	NAE (100)	AE (0.8)^^^	4.8	507
A2	M	Cau.	33	Acute	29d	TY8 (Nef 128-135; B*3501)	AE (100)	AE (1.3)	6.9	253
A3	M	AA	29	Acute	23d	RF10 (Nef 134-143; A*2301)	NAE (100)	NAE (0.5)	7.0	437
A4	M	Cau.	37	Acute	92d	RF10 (Nef 134-143; A*2301)	AE (100)	AE (0.9)	5.7	575
C1	F	Cau.	40	Chronic	4y	TL10 (Nef 128-137: B*07)	NAE (100)	AE (1)	5.0	405
C2^~^	M	AA	54	Chronic	8y	TL10 (Nef 128-137: B*07)	AE (100)	AE (-3.2)^@^	3.9	539
				Chronic		KY11 (Nef 105-115; C*07)	NAE (100)	–	3.9	685
C3	M	Cau.	37	Chronic	19y	TL10 (Nef 128-137: B*07)	AE (100)	–	4.5	228
C4	M	AA	55	Chronic	Unknown	RF10 (Nef 134-143; A*2301)	NAE (100)	NAE (1.8)	5.0	224
C5	M	Unknown^&^	3.3	Chronic	3y	KY11 (Nef 105-115; C*07)	NAE (100)	–	3.9	1331
C6	F	Unknown^&^	25	Chronic	Unknown	KY11 (Nef 105-115; C*07)	NAE (100)	–	2.5	968
C7	F	Unknown^&^	33	Chronic	Unknown	KY11 (Nef 105-115; C*07)	AE (84)	–	3.7	434

^*^ All subjects except C4 were treatment naive at the timepoint used to sort cells (subjects A1 and A4 started treatment at the timepoint used to sort cells); for acute HIV-infected subjects the transmitted founder virus was the same as the autologous virus at the timepoint used to sort cells. ^~^Samples tested with the TL10 and KY11 peptides and/or HLA class I tetramers were collected at different timepoints; ^&^ Haitian origin; ^#^ Determined by next-generation sequencing; ^$^ Determined by Sanger sequencing; ^@^ Sequenced prior to sorting timepoint; ^^^ Sequence is a mixture; - data not available; M, male; F, female; Cau., Caucasian; AA, African American; y, years; NAE, non-adapted epitope; AE, adapted epitope.

### Overlapping TCR Repertoire and Similar T Cell Clonality for NAE- and AE-Specific CD8^+^ T Cells in Chronic HIV Infection

Initially, the study aimed to compare the TCR repertoire of NAE- and AE-specific CD8^+^ T cells (**Figure 1B** scenario 1) reactive to the epitopes listed above. Two methods were utilized to ascertain antigen-specific CD8^+^ T cells in three subjects for TL10 and one subject for RF10 with chronic HIV infection: 1) HLA class I tetramers loaded with either the NAE or AE form of the epitope (‘resting’ CD8^+^ T cells); and 2) activation induced marker (AIM) expression after peptide stimulation (‘activated’ CD8^+^ T cells). Here, we examined chronic HIV infection as it represents long-term exposure to either the NAE or AE form and the subsequent evolution of the CD8^+^ TCR repertoire.

**Figure 2:**
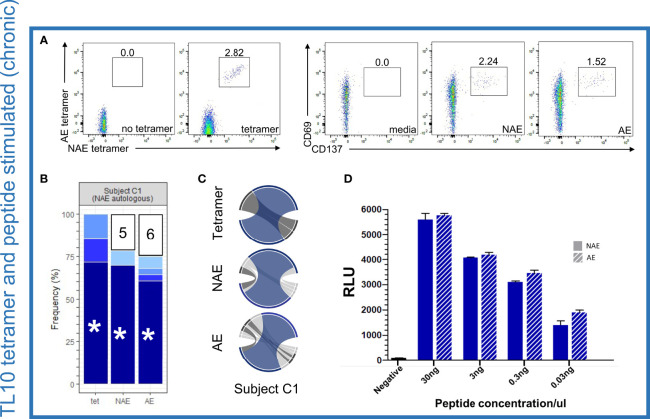
Dominant TCR α/β combinations for TL10-specific CD8^+^ T cells are cross-reactive for the NAE and AE form but show no difference in antigen-specific reactivity or sensitivity to the different forms of the HLA-peptide complex. **(A)** Flow cytometry plots for NAE- and AE-specific CD8^+^ T cells indicate a cross-reactive population using HLA class I tetramers and similar proportions following peptide stimulation for subject C1 for the epitope TL10 (negative controls included). **(B)** Common TCR CDR3 α/β combinations were observed in all three conditions with the remaining combinations unique to each condition grouped with the number of combinations listed (each color represents a single CDR3 combination). **(C)** Circos plots depict the TCR α/β CDR3 combinations observed in each condition with the CDR3 combination tested in panel D colored to match those depicted in panel B (highlighted with an asterisk). Note the circus plot shows the α and β chain of the highlighted combination are rarely observed with other alternate chains. The remaining TCR α/β CDR3 combinations common to all conditions are highlighted in dark grey. β chains are depicted on the bottom, with α chains depicted on the top. The width of each band correlates with the frequency of the respective α/β combination. **(D)** There was no difference in antigen-specific reactivity observed in the peptide dilution series (duplicates) of a common dominant TCR α/β CDR3 combination in subject C1 (indicated with an asterisk in panel B) for the NAE and AE form of TL10 using a T cell reporter assay. The negative control is transfected Jurkat cells with antigen presenting cells minus peptide. RLU = relative light units. N = 1.

High resolution TCR sequencing at the complementarity-determining region 3 (CDR3) level was performed on single cell sorted resting dual tetramer^+^ and activated CD8^+^ T cells (see *Materials and Methods* for details). This region of the TCR is the main site of interaction with the HLA-peptide complex and defines antigen specificity. Flow cytometry analysis of tetramer sorted cells revealed only dual tetramer^+^ (cross-reactive) CD8^+^ T cells for the epitope TL10 in subject C1 (**Figure 2A**), consistent with our prior study (15). The same cross-reactive dual tetramer^+^ pattern was observed for subjects C2 and C4 for the epitopes TL10 and RF10, respectively (**Figures S1A, B**). For confirmation of the cross-reactive nature of the TCR repertoire and ability of both assays to detect antigen-specific T cells at similar frequencies, CD8^+^ T cells from subject C1, C2, and C4 were activated separately (AIM assay as measured by the expression of activation markers CD69 and CD137) by peptides representing either the NAE or AE form of the relevant epitope (22) (**Table S1**). We observed a large overlap between the TCR repertoire of the dual tetramer^+^ and NAE- or AE-activated CD8^+^ T cells, including for specific TCR α/β combinations (**Figures 2B, C**). Furthermore, there was a high level of CDR3 overlap in all conditions for subjects C2 and C4 (**Figures S1C, D** and **Table S2**). While HLA class I tetramers were not available for the epitope KY11, four subjects tested with peptides for this epitope also showed overlapping TCR repertoires for NAE- and AE-activated CD8^+^ T cells (**Table S2**).

As a formal comparison of the TCR repertoires, TCR clonality (measure of the proportion of unique clonotypes of the total repertoire) was examined but there was no difference between the NAE and AE peptide activated CD8^+^ T cells for the epitopes tested in the chronic HIV-infected subjects and no difference in clonality between chronic and acute HIV-infected subjects (**Figure S2**; although there was a slight trend for higher clonality in chronic HIV infection, p=0.13). Overall, these results strongly suggest an almost complete overlap of the CD8^+^ TCR repertoire recognizing the NAE and AE form of TL10, RF10 and KY11 epitopes for all chronic HIV-infected subjects tested. TCR sharing and similarities in clonality between NAE- and AE-activated CD8^+^ T cells was not influenced by T cell memory subtype (**Figure S3**) or autologous form of the epitope. These results are consistent with our previous research that utilized low-resolution flow cytometry to show no difference in TCR Vβ-chain family expression levels between NAE- and AE-activated memory CD8^+^ T cells (14). Of note, TCRs may belong to the same Vβ-chain family but have different CDR3s and therefore antigen specificity reflecting the low resolution analysis of the earlier study (14).

### No Difference in Reactivity to the NAE and AE Form of Epitopes for Cross-Reactive TCRs Identified in Chronic Infection

Given the cross-reactive nature of the NAE- and AE-specific CD8^+^ T cell response, we sought to determine if there was a difference in the antigen specific reactivity to NAE or AE forms of an epitope (**Figure 1B** scenario 2), as had been suggested by our previous work (14, 15). A TCR reporter assay (23) was utilized to assess the reactivity of TCR α/β combinations cross-reactive to the NAE and AE form of the epitopes TL10 and RF10 identified in the chronic HIV-infected individuals. In this assay, specific TCR α/β combinations were co-transfected with CD8α into a Jurkat T cell line for co-culture with the relevant HLA class I allele expressing antigen-presenting cells pulsed with either the NAE or AE peptide at different peptide titrations to compare binding strengths (reactivity) of the TCR-peptide-HLA complex. There was no difference in the reactivity of a dominant cross-reactive TCR α/β combination for the NAE and AE form of the TL10 epitope in subject C1 (**Figure 2D**). Similarly, common but sub-dominant cross-reactive TCR α/β combinations for subjects C2 and C4 (epitopes TL10 and RF10, respectively) showed no difference in reactivity to the NAE and AE form of the epitope (**Figures S1E, F**).

### Loss of Polyfunctional Potential in Cross-Reactive ‘Resting’ Tetramer^+^ CD8^+^ T Cells in Subjects With the AE Autologous Form of the Epitope

**Figure 3:**
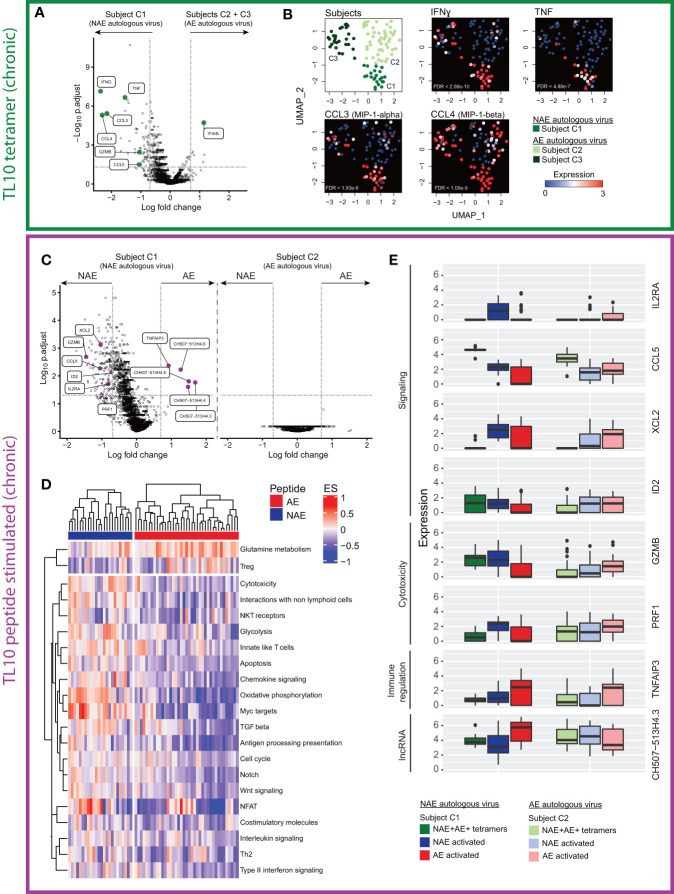
Single cell transcriptome analysis of cross-reactive resting (dual tetramer^+^) and activated (peptide stimulated) memory CD8^+^ T cells for the epitope TL10 reveals distinct profiles dependent on NAE/AE status of autologous HIV epitope. **(A)** Volcano plot of cross-reactive resting memory CD8^+^ T cells to the epitope TL10 demonstrates a number of significant DEGs across donors with different autologous viruses (N = 3). Key effector genes (pro-inflammatory, chemokine and cytotoxic) are highlighted in green. Horizontal and vertical dotted lines represent a significant threshold of a false discovery rate (FDR) of 0.05 and a fold change (natural log) of |2|, respectively. Note XIST (highest point) is highly expressed in subject C1 relative to the other subjects, as C1 is female while C2 and C3 are male. **(B)** UMAP representation of cross-reactive resting memory CD8^+^ T cells for subjects C1-3 with heatmap expression levels of significant DEGs suggestive of polyfunctional and cytotoxicity capacity from panel A (N = 3). **(C)** Volcano plots of cross-reactive activated memory CD8^+^ T cells in subject C1 showed significant DEGs after NAE and AE peptide stimulation, but no significant DEGs were observed for subject C2 (N = 2). Effector genes driving multiple biological pathways (in D) are highlighted in purple. **(D)** Pathway analysis of the transcriptome of the cross-reactive activated memory CD8^+^ T cells in subject C1 shows different pathways were upregulated depending on the form of the stimulating peptide (N = 1). Outcomes from hierarchical clustering are indicated. Only pathways that have a significant difference between stimulations (p<0.05) are depicted. Each column is one cell and horizontal bars indicate the stimulating peptide. **(E)** Box plots demonstrate the fold change in expression level of significant DEGs in multiple pathways in panel D in the cross-reactive resting (dual tetramer^+^) and activated memory CD8^+^ T cells in subjects C1 and C2 (N = 2).

We then examined the transcriptome profile of the cross-reactive (dual tetramer^+^) ‘resting’ CD8^+^ T cells with respect to the autologous virus (**Figure 1B** scenario 3). For the TL10 epitope, we had access to samples from three chronic HIV-infected subjects (C1-C3) that had either the NAE or AE form of the epitope in their viral quasispecies at the time of single cell sorting (**Table 1**). Data from subject C1 (autologous NAE form) was compared to the combined data from subjects C2 and C3 (autologous AE form). There were 67 statistically significant differentially expressed genes (DEGs) between the two groups of cross-reactive ‘resting’ TL10-specific CD8^+^ T cells; 61 genes were upregulated in subject C1 with the NAE autologous form and six genes were upregulated in subjects C2 and C3 with the AE autologous form (**Figure 3A**). In subject C1, the top ten genes with the highest fold change difference included key genes that encode anti-viral effector molecules. These genes included IFNɣ, TNFα, and chemokines RANTES (CCL5), MIP-1α (CCL3), and β (CCL4), which have been shown to be associated with enhanced HIV control (24), and GZMB that encodes the cytotoxic molecule granzyme B. Furthermore, uniform manifold approximation and projection (UMAP) analysis showed, for the most part, high co-expression of these genes in the cross-reactive tetramer^+^ ‘resting’ CD8^+^ T cells in subject C1 (**Figure 3B**). Of note, subject C1 showed evidence of transition to the AE form within one year of this sampling timepoint, suggestive of strong immune pressure on the TL10 epitope at the time of sorting. In contrast, subject C2 had maintained the AE form of TL10 over a period of three years (unknown if they ever had the NAE form) and subject C3 had been infected with HIV for approximately 19 years prior to the sorting timepoint (**Table S3**). These results suggest that the resting tetramer^+^ CD8^+^ T cells in a subject with only the NAE form of the epitope (and evidence of strong immune pressure leading to adaptation at a later timepoint) are in a more “ready” state with an increased polyfunctional potential (ability to produce multiple cytokines at the same time) to develop a more ‘effective’ anti-HIV immune response.

### Changes in Transcriptome Profile of Cross-Reactive CD8^+^ T Cells Can Be Associated With the AE Form of Epitope

Although these cross-reactive CD8^+^ T cells recognize both NAE and AE forms of an epitope regardless of autologous virus, they could still be functionally different post-stimulation by different peptides (15). Thus, we sought to examine the effect of stimulation with peptides representing the NAE and AE form of TL10 on the transcriptome of cross-reactive ‘activated’ CD8^+^ T cells for subjects C1 and C2 (subject C3 had insufficient cells to examine the effect of peptide stimulation). For subject C1 (autologous NAE form), there were 156 significant DEGs between ‘activated’ CD8^+^ T cells following NAE and AE peptide stimulation, but no significant DEGs for the two populations of activated cells for subject C2 (autologous AE form; **Figure 3C**). However, of the 156 significant DEGs for subject C1, only five of these genes were significantly upregulated after AE peptide stimulation: the anti-inflammatory gene TNFAIP3 and a family of long non-coding RNA (lncRNA; CH507-513H4.3, CH507-513H4.4, CH507-513H4.5 and CH507-513H4.6) (**Figure 3C**). Note, the lncRNAs (designated as CH507.513HX) are paralogs and the multi-mapping analysis approach for sequence reads adopted here means that one or more of these RNA species are present and require further validation to confirm which form(s) are expressed in the cells. Furthermore, ribosomal RNA cannot be excluded as contributing to the expression levels of these lncRNAs due to sequence overlap at the chromosomal location, however cells with ribosomal content above a specified threshold were excluded from analyses (see *Materials and Methods* for details).

A pathway analysis of the transcriptomes from these two populations of cells within subject C1 revealed several pathways over-represented in the NAE-stimulated cells, including cytotoxicity and chemokine signalling (**Figure 3D**). Genes contributing to each pathway were identified and those common to multiple pathways were then compared across the two subjects C1 and C2 for both cross-reactive resting (dual tetramer^+^) and activated (peptide stimulated) CD8^+^ T cells (**Figure 3E**). Genes such as GZMB and CCL5, which have been shown to be critical to cytotoxic or non-cytotoxic anti-HIV CD8^+^ T cell responses, respectively (24, 25), were significantly different in two comparisons: 1) resting dual tetramer^+^ CD8^+^ T cells between subjects with the NAE (subject C1) and the AE (subjects C2 and C3) form of TL10 (**Figure 3A**); and 2) between the NAE- and AE-activated cross-reactive CD8^+^ T cells for subject C1 with the NAE form of TL10 (higher in NAE-activated cells; **Figure 3C**). This suggests that a subject’s autologous form of TL10 can influence the transcriptome of epitope-specific CD8^+^ T cell responses, with the NAE autologous form associated with a more ‘effective’ immune response.

As T cell exhaustion has been associated with ineffective T cell responses and chronic antigen stimulation (26), specific markers associated with exhaustion (such as PD-1, LAG3, CTLA-4 and transcription factor EOMES) were examined across the different conditions for subjects C1-C2 (**Figure S4**). There were no significant differences in the expression of exhaustion markers in the cross-reactive resting or NAE- and AE-activated CD8^+^ T cells for subjects C1 and C2. Although these exhaustion markers were not examined at the protein level in the flow cytometry panel used in this study, we have observed a correlation between protein and transcript expression for these genes in another experiment using the CITE-seq technique that can simultaneously capture protein and RNA from single cells (**Figure S5**).

Overall, cross-reactive CD8^+^ T cells from chronic HIV-infected individuals showed transcriptome differences that were associated with the autologous form of the epitope, and likely predicts the strength of the host’s immune pressure on the epitope. Specifically, ‘resting’ and ‘activated’ CD8^+^ T cells from subjects with the NAE form of the epitope appeared to have a more ‘effective’ anti-HIV immune response as indicated by the increased expression of genes reflecting polyfunctional potential and cytotoxicity pathways.

### Single-Reactive ‘Resting’ CD8^+^ T Cells Are Suggestive of a More ‘Effective’ Immune Response When an Individual Has the NAE Autologous Form of the Epitope in Acute Infection

After observing transcriptomic differences based on the autologous form of epitopes in chronic HIV-infected subjects, we considered if this was a phenomenon specific to chronic infection. Thus, we screened a cohort of acute HIV-infected subjects according to their transmitting founder virus (TFV) sequence and HLA class I repertoire. We identified four subjects, two with antigen-specific CD8^+^ T cell responses to TY8 and two to RF10. Furthermore, each set of subjects was infected by a TFV encoding either the NAE or AE form of the epitope (**Table 1**). We next sought to determine whether similar patterns of cross-reactivity and functionality of antigen-specific CD8^+^ T cells observed in chronic HIV-infected subjects were also observed in subjects during the acute phase of HIV infection in cases where the TFV is known. Here, we were able to examine the antigen-specific CD8^+^ T cell responses in two acute HIV-infected subjects (A1 and A2) that had either the NAE or AE form of the epitope TY8 (**Table 1**). The founder virus for subject A1 had the NAE form of TY8 that then transitioned to the AE form within the first year of infection. As suggested above for subject C1, this indicates strong immune pressure on this epitope. The founder virus for subject A2 was the AE form and this had not changed more than one year from infection (**Table 1**).

**Figure 4:**
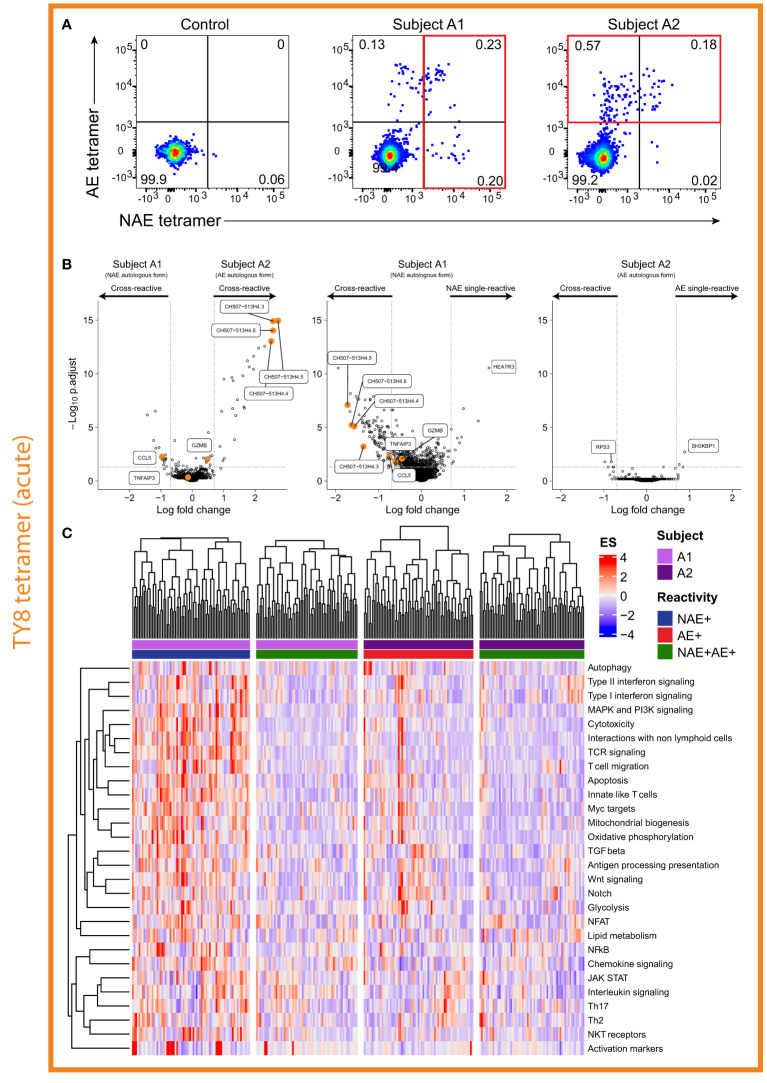
Single-reactive CD8^+^ T cells in individual with the NAE form of epitope appears to produce a more ‘effective’ immune response. **(A)** Flow cytometry plots for acute HIV-infected subjects A1 and A2 revealed both cross- and single-reactive CD8^+^ T cells for the epitope TY8. **(B)** Volcano plots comparing single- and cross-reactive cells for subjects A1 and A2 revealed differences in the number of significant DEGs based on the autologous form of TY8. Select DEGs observed between cross-reactive resting CD8^+^ T cells and between NAE and AE stimulated CD8^+^ T cells in the chronic HIV-infected subjects – i.e. GZMB, CCL5, TNFAIP3 and the CH507 lncRNA family, are highlighted in the volcano plots. Horizontal and vertical dotted lines represent a significant threshold of an FDR of 0.05 and a fold change of |2|, respectively. **(C)** Pathway analysis of single- and cross-reactive CD8^+^ T cells for subjects A1 and A2 revealed a number of significantly different pathways between the NAE single-reactive CD8^+^ T cells of subject A1 and the other three CD8^+^ T cell populations (N = 2).

Contrary to the observations in chronic HIV-infected subjects, HLA class I tetramer staining of subjects A1 and A2 demonstrated the existence of both cross-reactive and single-reactive tetramer^+^ ‘resting’ CD8^+^ T cell populations for the epitope TY8 (**Figure 4A**). Due to limited cell availability, only one single-reactive and the cross-reactive cell population were sorted with priority given to the single-reactive population matching the autologous virus at the time of sorting for each individual. High resolution TCR sequencing confirmed no TCR α/β overlap between single- and cross-reactive tetramer^+^ ‘resting’ CD8^+^ T cells in subject A1 but some overlap between single- and cross-reactive ‘resting’ CD8^+^ T cells in subject A2 (**Table S2**). However, it is important to note that the gating for subject A2 may have included some overlap between these two populations (**Figure 4A**). Unfortunately, indexed sorting was not completed on this subject and back-gating for specific T cells could not be performed.

As for chronic HIV infection, here we initially compared the transcriptomic profiles of ‘resting’ CD8^+^ T cells based on the autologous virus of the subject. Thirty-nine DEGs were identified between the cross-reactive ‘resting’ dual tetramer^+^ CD8^+^ T cells of acute HIV-infected subjects A1 and A2 (**Figure 4B**). Within a subject, for the single- and cross-reactive ‘resting’ CD8^+^ T cell populations, a large number of significant DEGs were observed to be upregulated in the single NAE-reactive CD8^+^ T cells for subject A1 with the NAE autologous form (126 genes), while only a handful of significant DEGs (8 genes) were observed for the two populations in subject A2 with the AE autologous form of TY8 (**Figure 4B**). This is similar to the chronic HIV-infected subjects, where no significant DEGs were observed for subject C2 who had the AE form of TL10.

We next sought to compare these results with those in chronic HIV infection. Of the significant DEGs identified in the chronic HIV-infected subjects thought to be associated with a more ‘effective’ immune response (CCL3, CCL4, CCL5, GZMB, IFNɣ and TNFα; **Figure 3**), CCL5 was the only significant DEG observed (although GZMB reached significance by p-value, it did not by fold change). As with the chronic HIV-infected subjects for TL10, CCL5 was significantly upregulated in conditions we predict to have a more ‘effective’ immune response; the cross-reactive and single (NAE)-reactive cells of subject A1 with the NAE autologous form (**Figure 4B**). Of the significant DEGs identified in the chronic HIV-infected subjects thought to be associated with a more ‘ineffective’ immune response (lncRNAs, TNFAIP3), the lncRNAs were upregulated in the cross-reactive cells of subject A2 (AE autologous form; supporting the chronic HIV-infection results), but were also significantly upregulated in the single-reactive (NAE-reactive) cells of subject A1 (along with CCL5). These contradictory results suggest that these lncRNAs may not be a good marker of an ‘ineffective’ immune response and furthermore highlight the likely complexity of these lncRNAs in immune modulation.

To gather biological insights into the transcriptome profiles of the different CD8^+^ T cell populations, we performed a pathway analysis on the single- and cross-reactive resting memory CD8^+^ T cells from subjects A1 and A2 (**Figure 4C**). NAE single-reactive CD8^+^ T cells from subject A1 (NAE autologous virus) showed pathways more suggestive of an ‘effective’ immune response (cytotoxicity and apoptosis) than AE single-reactive CD8^+^ T cells in subject A2 and the cross-reactive T cells in both subjects. These results suggest that subject A1, with the NAE autologous form of the epitope TY8, appears to have two distinct populations of TY8-specific CD8^+^ T cells in the context of TCR repertoire (**Table S2**) and the transcriptome. The NAE single-reactive CD8^+^ T cells in subject A1 may be exerting greater immune pressure on HIV (‘effective’ immune response) and is the potential driving source for epitope adaptation (observed 10 months after sorting; **Table S3**) and may lead to subsequent selection of less effective cross-reactive CD8^+^ T cells.

**Figure 5:**
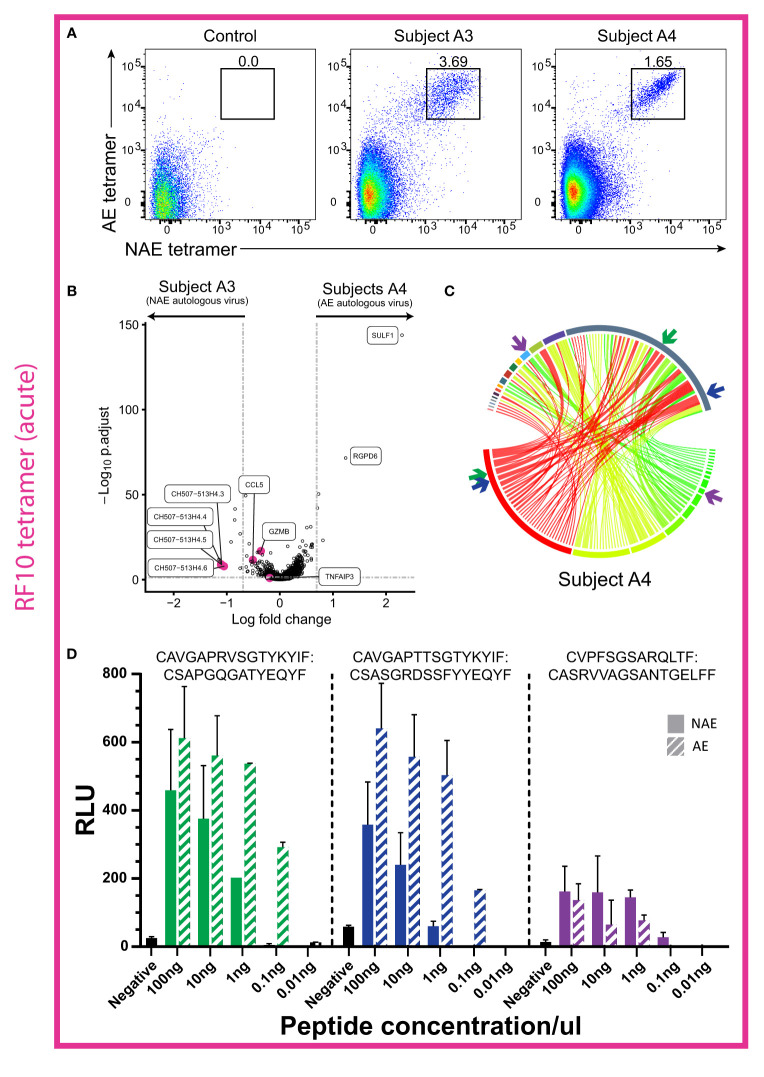
Cross-reactive CD8^+^ T cells in two acute HIV-infected individuals showed little difference in transcriptome profiles, but TCRs from one subject revealed a higher antigen-specific reactivity to the AE form of RF10. **(A)** Flow cytometry plots in acute HIV-infected subjects A3 and A4 reveal a distinct cross-reactive (dual tetramer^+^) CD8^+^ T cell population for the epitope RF10. **(B)** Volcano plot for resting cross-reactive (dual tetramer^+^) memory RF10-specific CD8^+^ T cells for subjects A3 and A4 revealed few significant DEGs (N = 2). Horizontal and vertical dotted lines represent a significant threshold of a FDR of 0.05 and a fold change of |2|, respectively. **(C)** Circos plot reveals no dominant TCR α/β combination in subject A4, but rather a high diversity of TCRs (N = 1). TCR α/β combinations are colored as a heatmap. β chains are depicted on the bottom, with α chains depicted on the top. The width of each band correlates with the frequency of the respective α/β combination. **(D)** Two of the three TCR α/β combinations tested in a TCR reporter assay showed higher antigen specific reactivity to the AE form of RF10 (subject A4; N = 1). The combinations tested are highlighted with colored arrows in panel **(B)** The negative control is transfected Jurkat cells with antigen presenting cells minus peptide. RLU = relative light units. Note that RLU values cannot be compared across TCRs due to variability in plasmid transfection rates (see *Materials and Methods* for more details).

We next examined two additional acute HIV-infected subjects (A3 and A4) that had CD8^+^ T cell responses to RF10 and their TFV (and autologous virus) was either the NAE (subject A3) or AE (subject A4) form. Of note, unlike subject A1 above, subject A3 did not transition to the AE form of the epitope during the six months following sorting (**Table S3**), suggestive of weaker immune pressure on the epitope. Flow cytometry analysis revealed both subjects had only dual tetramer^+^ (cross-reactive) CD8^+^ T cells (**Figure 5A**). These results suggest complete overlap in the TCR repertoire for the NAE and AE form of the RF10 epitope, as observed for the chronic HIV-infected subjects.

Transcriptome analysis of cross-reactive resting CD8^+^ T cells (dual tetramer^+^) in the two subjects (A3 and A4; **Figure 5B**) revealed 12 genes significantly upregulated in subject A3 (NAE autologous form) and five genes significantly upregulated in subject A4 (AE autologous form; **Figure 5B**). The effector molecules GZMB and CCL5, that were upregulated in the chronic HIV-infected subject C1 with the NAE autologous form of TL10, had a significant p-value in cross-reactive CD8^+^ T cells in subject A3 (NAE autologous form), however the fold change was below the threshold of |2|. The lncRNAs identified in the chronic HIV-infected subjects were also upregulated in subject A3, but not in conjunction with the immune-suppressive marker TNFAIP3. These results suggest that subject A3 (NAE autologous form) may exhibit a more ‘effective’ immune response than subject A4; albeit with low efficacy as the immune response was unable to induce the NAE to AE transition in the autologous virus.

Interestingly, three TCR α/β combinations specific for TY8 from the cross-reactive CD8^+^ T cells in subject A4 (**Figure 5C**) were tested in the TCR reporter assay to assess any differences in antigen-specific reactivity (**Figure 1B** scenario 2). Two TCR α/β combinations revealed a higher antigen-specific reactivity to the AE form of RF10 (**Figure 5D**). These results suggest that in acute HIV infection, cross-reactive TCRs may have differing antigen-specific reactivity to the NAE and AE form that may influence the selection of TCRs observed in chronic HIV infection. Furthermore, although we do not have the power to detect a significant difference in TCR clonality between acute and chronic HIV-infected subjects (dual tetramer^+^ cells only), there was a mean difference of 0.31 (higher clonality in chronic subjects; **Figure S2B**).

### Distinct T Cell Gene Sets Highlight Effector Potential in ‘Resting’ (Tetramer^+^) and ‘Activated’ (Peptide Stimulated) CD8^+^ T Cells for Both Acute and Chronic HIV-Infected Subjects

**Figure 6:**
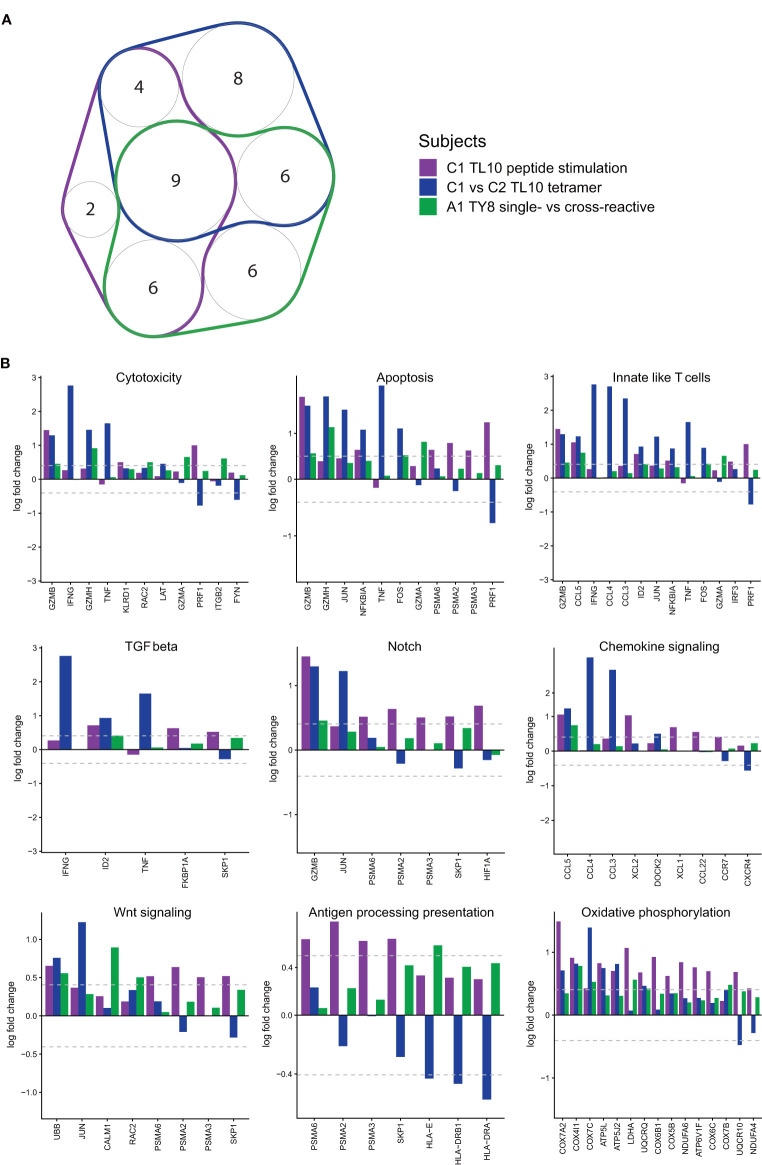
Pathway analyses reveal common upregulated pathways and genes in antigen-specific CD8^+^ T cells likely exhibiting an ‘effective’ immune response. GSVA pathway scores were used to measure cell expression of genes in specific pathways (Nanostring CAR-T). **(A)** A number of pathways with a significant difference (p<0.05; Kruskal-Wallis) were common in the different conditions (N = 3). Only pathways with three or more genes were taken through for further analysis (memory markers and TCR diversity were excluded). **(B)** The nine pathways that were common to all categories were taken through to determine potential driving genes (N = 3). All conditions are shown, but only genes that have at least one significant fold change (≥|1.5|) were included.

We next sought to delineate common pathways characteristic of ‘effector’ T cells by identifying shared gene set signatures common in resting and activated memory CD8^+^ T cells. In both chronic and acute HIV-infected subjects, differential gene expression and pathway analyses showed unique transcriptome patterns and T cell gene set expressions in resting and activated memory CD8^+^ T cells dependent on the autologous form of the epitope. To identify common pathways, we examined the following three comparisons that showed DEGs for those cell populations that we suggest from our prior experiments above to have an effective immune response against HIV (**Figure 6A**): i) dual tetramer^+^ ‘resting’ TL10-specific CD8^+^ T cells for subjects with the autologous NAE or AE form (chronic HIV infection; subject C1 versus subjects C2 and C3); ii) NAE versus AE peptide stimulated ‘activated’ TL10-specific CD8^+^ T cells within subject C1 with the NAE form of the epitope (chronic HIV infection); and iii) single- versus cross-reactive ‘resting’ TY8-specific CD8^+^ T cells for subject with the autologous NAE form (acute HIV infection; subject A1). Interestingly, eight out of nine gene sets that were common between the three comparisons were enriched in NAE activated CD8^+^ T cells and in resting CD8^+^ T cells in subjects with the NAE form of the virus (**Figure 6B**; antigen processing presentation was the exception). Enriched gene sets represent several distinct T cell functional pathways in effector CD8^+^ T cells, including activation (oxidative phosphorylation), proliferation (Notch signalling and Wnt signalling) and CD8^+^ T cell effector functions (cytotoxicity and chemokine signalling). Not surprisingly, these same T cell populations were found to express genes likely to represent innate-like T cells known to elicit robust effector responses (27).

From the common effector gene set signatures, we found overlapping anti-viral and cytotoxic genes that have been previously identified in the differential gene expression analyses that appear to be driving these pathways. Genes with a significant fold change (≥|1.5|) in at least one comparison were plotted for all nine pathways that overlapped for all comparisons (**Figure 6B**). As we aimed to identify overlaps in genes driving significant pathways, a fold change of |1.5| was selected here, as opposed to the stricter fold change of |2| used to identify DEGs. Genes associated with enhanced HIV control identified in the analysis of subjects C1-C3 for the epitope TL10 (IFNɣ, TNF, CCL3 and CCL4) were identified as drivers in multiple biological pathways such as cytotoxicity, chemokine signalling and innate-like T cell signatures. Similarly, GZMB was a significant driver of the cytotoxicity and notch signalling pathways. These genes occurred at higher levels across subjects with a predicted ‘effective’ immune response. Consequently, it appears an ‘effective’ immune response can broadly be defined by an NAE autologous virus that exerts sufficient pressure to adapt to the AE form at a later timepoint. Subsequently, common pathways can be identified within these conditions that appear to be driven by a handful of genes, a number of these genes are highlighted as significant DEGs throughout the analyses (GZMB, IFNɣ, TNF, CCL3 and CCL4).

## Discussion

### Extensive Cross-Reactivity of CD8^+^ T Cells Recognizing the NAE and AE Form of Select HIV Epitopes

As immune control of several pathogens is increased with a diverse TCR repertoire (20, 21), our first proposed mechanism of non-classical adaptation was a reduction in the diversity of TCR repertoire recognizing the AE form of an epitope (**Figure 1B** scenario 1). However, this was not the case, with all chronic HIV-infected subjects tested demonstrating complete cross-reactivity of TCRs for the NAE and AE form of the epitope. This lack of differentiation in the TCR repertoires of NAE- and AE-specific CD8^+^ T cells in chronic HIV infection extended to the antigen-specific reactivity of the cross-reactive CD8^+^ T cells (both dominant and sub-dominant) to the two forms of the epitope. This result was somewhat surprising given our previous results (14) indicating higher functional avidity (based on IFNɣ release) of the AE form of epitopes that exhibit non-classical adaptation (**Figure 1B** scenario 2).

In contrast, for the acute HIV-infected subjects A1 and A2, there were clear populations of single-reactive as well as cross-reactive CD8^+^ T cells recognizing the epitope TY8, indicating the presence of NAE- or AE-specific. The lack of overlap in the TCR repertoire of the two populations suggests a selection process may occur between acute and chronic HIV infection. Interestingly, although the acute HIV-infected subjects A3 and A4 demonstrated complete cross-reactivity for the epitope RF10, as seen for the chronic HIV-infected subject C4 for the same epitope, two of three TCR α/β combinations identified in subject A4 with the AE form of the epitope showed higher antigen-specific reactivity towards the AE form of RF10. These results suggest that differences in antigen-specific reactivity can occur due to a single amino acid change and that viral adaptation has the potential to alter downstream TCR signalling *via* differences in TCR-HLA-peptide reactivity, a mechanism that has been described elsewhere (28), such as the more general altered peptide ligand model (12): a similar phenomenon to non-classical adaptation in which antigen-derived peptides that vary by one amino acid have varied abilities to stimulate T cell functions. Changes described by the altered peptide ligand model can lead to differences in affinity for the presenting HLA molecule, altering TCR recognition through modifications to the TCR-peptide-HLA complex (13) and may represent the observed changes to epitopes containing a non-classical adaptation. Furthermore, there may be selection of cross-reactive TCRs from acute to chronic HIV infection, a potential viral adaptation strategy. The increase in TCR repertoire clonality from acute to chronic HIV infection supports this model.

### Cross-Reactive ‘Resting’ and ‘Activated’ CD8^+^ T Cells Exhibit Different Transcriptome Profiles Based on a Subject’s Autologous Virus

Transcriptome analyses were utilized to assess different immune profiles based on a subject’s autologous virus (**Figure 1B** scenario 3). For TL10-specific ‘resting’ CD8^+^ T cells, there was a clear signal of polyfunctional and cytotoxicity potential (upregulation of IFNɣ, TNFα, CCL3, CCL4, CCL5 and GZMB) in subject C1 with the NAE autologous form of the virus, which was not seen in subjects C2 and C3 (AE autologous form). Polyfunctional T cells have been associated with a more effective immune response (29, 30), with a loss/reduction in polyfunctionality shown to reduce immune effectiveness in various infections, including HIV (31). Subject C1 transitions to the AE form of TL10 at a later timepoint after sorting, suggesting that an effective polyfunctional immune response may induce this adaptation over time. Subjects C2 and C3, with the AE autologous form did not have this polyfunctional transcriptomic profile. There was insufficient numbers of individuals followed longitudinally to determine the effect of duration of HIV infection on the transcriptomic effect of these epitope changes (**Table 1**), but it is known that the time course of HIV adaptations to several epitopes (including classical adaptation) can vary considerably (32, 33). Furthermore, while these adaptations have been identified at the population level, it is possible that other factors not assessed here such as other host immunogenetic variants including HLA, KIR and ERAP genotypes and compensatory mutations in the virus may impact the presence or absence of these adaptations.

**Figure 7:**
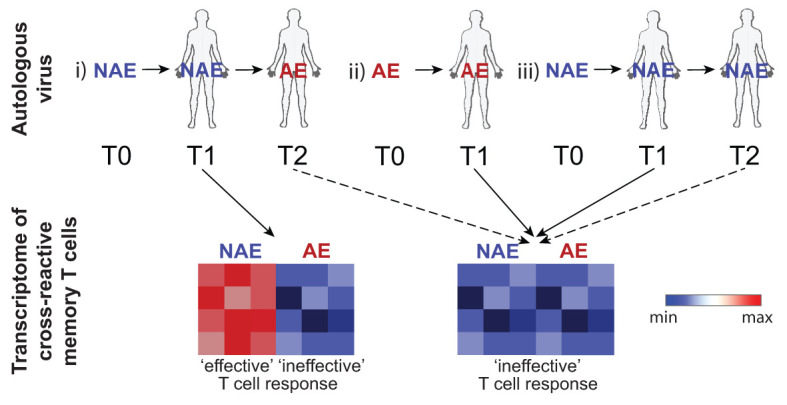
Schematic depicts the transcriptome of cross-reactive memory T cells following peptide stimulation based on the TFV (T0), autologous (T1-2) virus at time of sampling and any future transitions. In an individual where the NAE form transitions to the AE form over time (reflecting strong immune pressure; scenario i), cross-reactive memory T cells that have not ‘seen’ the AE form of the epitope (samples tested at timepoint T1) will exhibit an RNA transcriptomic profile dependent on the stimulating peptide form with activation by the NAE form reflecting features consistent with an ‘effective’ immune response. Note that timepoint T2 in this scenario was not able to be tested. Cross-reactive memory T cells previously activated by the autologous AE form of the epitope (scenario ii) at timepoint T1 would have an RNA transcriptomic profile reflective of an ‘ineffective’ response irrespective of stimulating peptide form. Similarly, cross-reactive memory T cells that retain the NAE form at all timepoints (scenario iii) may also show a transcriptomic profile reflective of an ‘ineffective’ response as there was not sufficient immune pressure to drive the NAE form to the AE form. Dotted lines indicate scenarios not tested. Blue indicates down-regulation of transcripts associated with an effective immune response.

A comparison of NAE- and AE-stimulated ‘activated’ CD8^+^ T cells for TL10 only revealed significant DEGs for subject C1 and not for subject C2 (NAE versus AE autologous forms, respectively). After NAE stimulation of TL10, subject C1 also exhibited genes associated with an enhanced HIV response such as CCL5 and GZMB (25), involved in pathways for activation, proliferation and cytotoxicity. In contrast, after AE stimulation, cells from subject C1 showed upregulation of TNFAIP3 and a family of lncRNAs. TNFAIP3 encodes for the zinc-finger ubiquitin-editing enzyme A20, which has the capacity for potent anti-inflammatory regulation by restricting NFkB dependent signaling (34). Consequently, the increase in TNFAIP3 observed after AE stimulation may negatively impact the immune response of individuals with the AE autologous form of TL10. The specific function of the lncRNAs identified in this comparison, and indeed in others, is not clear but there is evidence that lncRNAs in general are involved in controlling cell differentiation, cell cycle and immune processes (35). While there were insufficient cells to perform detailed functional assays, the robust single cell transcriptome data suggests that a single amino acid adaptation in HLA-restricted HIV CD8^+^ T cell epitopes may modulate immune responses. Furthermore, our previous work has indicated AE-specific CD8^+^ T cells show lower polyfunctionality (36). The results from the analysis of the cross-reactive CD8^+^ T cell responses are summarised as a model in **Figure 7**.

### Overlapping T Cell Effector Pathways Identified in Antigen-Specific CD8^+^ T Cells in Both Acute and Chronic HIV Infection

Common T cell pathways (namely cytotoxicity and apoptosis) were observed in subjects with an ‘effective’ immune response (NAE form that transitioned to the AE form at a later timepoint) in both acute and chronic HIV infection, although the genes driving these differences varied (for example, cytotoxicity was driven by GZMB, IFNG, GZMH and TNF for subject C1 and C2 tetramer comparison, but by GZMB, KLRD1 and PRF1 for subject C1 peptide comparison). With respect to HIV control and an ‘effective’ immune response, the overarching determinant is having the NAE form of an epitope with enough immune pressure on that epitope to eventually drive it to escape to the AE form. Following this, subjects who fulfil this pre-requisite have pathways for cytotoxicity and apoptosis upregulated, with genes such as GZMB and CCL5 driving these pathways. As GZMB and CCL5 have previously been described in anti-HIV responses (24, 25), the analysis described here identified transcriptomic signatures of an ‘effective’ anti-HIV immune response. Identifying a number of these genes that overlap across pathways may prove useful in designing panels to test ‘effective’ immune responses following vaccination.

T cells chronically exposed to antigen typically express one or more markers indicative of immune exhaustion (26). Immune exhaustion results in a decreased ability to proliferate, reduced capability of producing cytokines, reduced cytolytic potential and an altered transcriptional profile (26). Exhausted T cells retain suboptimal functions, but are unable to eradicate tumors or pathogens such as HIV (26). It has been suggested that adaptation in the chronic stage of HIV, SIV and hepatitis C virus could occur in response to pressure from exhausted T cells, despite their diminished ability to perform effector functions (26). HIV adaptations that do not lead to a loss of immune recognition (i.e. non-classical) may harness this mechanism and/or loss of polyfunctionality (**Figure 1B** scenario 3 and 3B). The increased polyfunctional potential of subject C1 relative to subjects C2 and C3, as observed in their dual tetramer^+^ memory CD8^+^ T cells (**Figure 3B**), demonstrate one mechanism that may be at play in these chronic HIV-infected subjects.

It is possible that AE-activated memory CD8^+^ T cells displayed reduced immune functionality due to T cell exhaustion. However, there was limited expression of canonical T cell exhaustion transcripts aside from LAG3, EOMES, CD160, and TIGIT (**Figure S5A**). Interestingly, these transcripts were predominantly found in NAE-activated memory CD8^+^ T cells in subjects with the NAE autologous form of TL10. The expression of these exhaustion surface markers may reflect acute induced activation due to peptide stimulation as reported in previous studies (37–39).

This study was not able to test cytotoxicity *in vitro*, but previous research by our group has suggested that CD8^+^ T cells expanded by AE epitopes exhibit a higher level of cytotoxicity against CD4^+^ T cells during chronic infection (15), somewhat contradictory to the pathway analyses conducted here. Furthermore, we showed that AE-activated CD8^+^ T cells could facilitate dendritic cell maturation and consequently, enhanced HIV *trans*-infection (15). This is another potential mechanism by which non-classical adaptation confers a viral adaptation advantage distinct from classical adaptation, although it is likely that this is not the sole mechanism conferring an evolutional advantage to the virus.

An alternative mechanism of adaptation not tested in this study is a shift in immunodominance. As all epitopes tested in this study are within Nef, it cannot be excluded that the adaptation present in these epitopes may have led to a shift in immunodominance. However, it has been shown that in the acute phase of HIV infection a high proportion of CD8^+^ T cell responses target Nef. Furthermore, TL10 and KY11 are two epitopes with adaptations commonly observed in chronic HIV infection and have been shown to elicit CD8^+^ T cell responses (14). Previous research has demonstrated that novel adaptations can draw the immune response to regions that can better tolerate change (i.e. Gag or Pol), eliciting a high avidity, but exhaustive, CD8^+^ T cell response (14). Furthermore, different TCR and transcriptomic patterns may be present for epitopes in different HIV proteins. Further study of other anti-HIV responses in these subjects are needed, both cross-sectional and longitudinal (where NAE and AE autologous forms can be assessed in the same subject and/or from acute to chronic HIV infection), particularly for subjects C1 and A1 in which the autologous virus for the TL10 and TY8 epitope, respectively, transitioned to the AE form at a later timepoint. It would be of interest to know if the functional change is restricted to AE-reactive CD8^+^ T cells or whether this is a global phenomenon that also affects cells specific for other HIV epitopes within the same donors, or even cytomegalovirus T cell epitopes.

The number of subjects and diverse clinical presentations were two main limitations of this study; subjects had to have the appropriate restricting HLA allele for the epitope, a response to the epitope and the relevant autologous form. Screening of subjects proved that subjects covering all these criteria were rare (see *Materials and Methods* for more detail). While it is possible that clinical characteristics such as viral load, CD4^+^ T cell count and length of infection may impact the phenotypes observed (37, 40), the findings are still relevant for vaccine design.

Although a limited number of subjects and epitopes were examined in this study, our findings could be informative for future research, which would incorporate larger cohorts to confirm the biological relevance of such distinguished pathways and transcriptomic patterns based on a subject’s autologous virus. Elucidating the mechanisms of viral adaptation are fundamental concepts for host-pathogen interaction that have important clinical implications for vaccine design, partly due to the increase in the CD8^+^ T cell response towards AE epitopes observed from acute to chronic HIV infection (15). Vaccine design approaches would benefit from a greater understanding of the ways in which adaptation in transmitting strains of HIV or those reactivated from latency can modulate the human immune response, and therefore what aspects of host immune responses are important for durable protection. While mosaic vaccine approaches aim to incorporate viral strains from many HIV clades, it may be reasonable to exclude adaptations where the AE form has become consensus as the effect of HIV adaptations on the immune response may determine effectiveness of both prophylactic and therapeutic vaccines.

## Materials and Methods

### Study Design

As indicated in **Figure 1**, the objective of this study was to examine HIV T cell epitopes for which there was evidence that adaptation did not reduce immunogenicity of the epitope. Subjects with IFNɣ T cell responses to these epitopes were selected for high-resolution TCR and RNA transcriptomic analysis. The following HIV T cell epitopes in Nef were examined (see **Table S4** for HLA Class I genotyping): HLA-B*07-restricted TL10 (Nef 128-137; adapted amino acid underlined - TPGPGI/VRYPL), HLA-C*07-restricted KY11 (Nef 105-115; KRQD/EILDLWVY), HLA-A*2301-restricted RF10 (Nef 134-143; RY/FPLTFGWCF) and HLA-B*3501-restricted TY8 (Nef 128-135; TPGPGI/VRY). These epitopes were selected as they have been previously described in independent studies (14, 16) to contain an HLA-associated HIV polymorphism within the epitope, and for TL10 and KY11 were described in the Keane et al. study describing increased functional avidity of adapted epitopes for select HIV epitopes (14). A large number of subjects (n = 165) were screened for responses to selected epitopes, with only a subset passing criteria for sorting epitope-specific T cells. For the epitope TL10, 10 chronic HIV-infected subjects carrying HLA-B*0702 were screened for responses with ICS with only four subjects showing a response. Three of these were sorted (subjects C1-C3) and the remaining subject had a response that was too low to sort sufficient numbers of cells. For the epitope KY11, 10 chronic HIV-infected subjects carrying HLA-C*0702 were screened for responses with ICS with five subjects showing a response. For the epitope TY8, 27 chronic infected subjects carrying HLA-B*3501 (from 65 subjects) were screened by ELISpot in our previous work (15) with only four subjects having a response to both the NAE and AE form, but none having a response over the cut-off of 200 spot forming units (SFU). Thirty-three acute HIV-infected subjects were screened by ELISpot for epitope-specific responses in our previous work (191 NAE and 178 AE epitopes) (subset of data published in (15)) with 47 NAE- and 22 AE-positive responses identified. Of these subjects, none showed a response in more than one individual to both the NAE and AE form. Four subjects were selected based on their transmitted founder virus (TFV) and ELISpot response (subjects A1-A4).

### Study Subjects

Seven subjects were sampled during chronic HIV-1 infection and all but one were anti-retroviral therapy (ART) naïve. Four subjects were sampled during acute HIV-1 infection and three were ART naïve and one subject received ART during the course of follow-up (**Table S3**). Samples for the acute HIV-infected subjects and subject C4 were obtained from University of Alabama at Birmingham, Centre for Aids Research, Network of Integrated Clinical Systems, with the remaining subjects obtained from Vanderbilt University Medical Center. PBMCs were purified from leukapheresed or whole blood by FICOLL density gradient separation and cryopreserved. HLA typing to 4-digit resolution was performed as previously described (14, 16, 41).

### PBMC Stimulation and Flow Cytometry

Cryopreserved PBMCs were thawed and stained with tetramers (National Institutes of Health Tetramer Core Facility, GA, USA) or activated overnight for approximately 18 hours with high purity peptides (95%; Schafer-N, CPH, Denmark) at a final concentration of 1µg/mL. SEB (Millipore) at 1µg/mL was used as a positive control. PBMCs were cultured in RPMI supplemented with 10% human AB serum, 10mM glutamine and 10mM HEPES. Briefly, cells were washed and approximately 5×10^6^ PBMCs/well were activated with peptides overnight (or no stimulation in the case of tetramers) at 37°C and 5% CO_2_ in 1mL co-stimulatory media containing 1µL/mL of anti-CD28 and anti-CD49d (for peptide stimulation only; BD Biosciences). Anti-CD49d and anti-CD28 are standard additions to cells during peptide incubation for measurement of intracellular cytokine expression; the original paper by Waldrop et al. (42) used anti-CD28 and these assays were subsequently optimized with the addition of anti-CD49d by Gauduin et al. (43, 44). While we did not fix cells and sorted cells based on expression of activation markers, we kept our stimulation protocol similar to prior protocols used for *in vitro* cell stimulation. Cells were washed twice with PBS and stained with fluorescently labelled antibodies. Initially, cells were stained with CCR7-BV421 and tetramers (for the tetramer condition only) for 20 minutes at 37°C. Cells were washed and stained with Live/Dead Fixable Aqua (ThermoFisher) as a viability marker for 10 minutes at room temperature. Cells were washed and stained with the antibodies CD14-V500, CD19-V500, CD3-Alexa Fluor 700, CD4-PcCCy5.5, CD8 FITC, CD69-APC, CD137-PE, CCR7-BV421, PD1-PE-Cy7 (BD Biosciences) and anti-CD45RO-PE-Texas red (Beckman Coulter) for 20 minutes at room temperature. Cells were washed and sorted with a BD FACS Aria III. Data were analysed with FlowJo 10.1 (TreeStar). Activated CD8^+^ T cells were defined as CD69^+^/CD137^+^ with at least one 96-well plate sorted for each condition used for TCR and RNAseq analyses. An example of flow gating is depicted in **Figure S6**. In this study, similar proportions of tetramer^+^ and CD69^+^/CD137^+^ CD8^+^ T cells were observed for subjects, highlighting the effectiveness of the activation markers to identify antigen-specific T cells (**Figure 2A** and **Figures S1A, B**).

### Single Cell Sorting and cDNA Conversion

Single cell RNA-sequencing methodology was adapted from two previously described protocols, Smart-seq2 and MARS-Seq (45, 46). Cells were sorted into a 96-well plate containing 3µL of lysis buffer inclusive of a ribo-nuclease inhibitor. Oligo DT primers coupled with well-specific unique molecular identifiers (UMIs) were added and the plate was incubated at 65°C for 5 minutes, followed by 1 minute on ice. A reverse transcription master mix was added to the plate and run on a thermocycler for 30 minutes at 50°C followed by 10 minutes at 80°C. A first round PCR was conducted using the KAPA HiFi HotStart ReadyMix (Roche, Basel, Switzerland) and barcoded IS cDNA Amp primers. Second round PCRs were conducted with either TCR or gene specific primers (see below for more detail).

### T Cell Receptor Sequencing

Following the first round PCR, equal volumes of all sorted wells were pooled prior to a second round nested PCR using template switch oligo (ISTSO) forward primers and barcoded reverse primers specific to the alpha and beta chains of the TCR. PCR products were purified using Agencourt AMPure XP (Beckman Coulter, CA, USA) and pooled equimolar. Indexed libraries were created for sequencing using Truseq adapters and quantified using the KAPA Universal qPCR Library Quantification Kit (Kapa Biosystems Inc., MA, USA), as per the manufacturer’s instructions. Samples were sequenced on an Illumina MiSeq using a 2×300bp paired-end chemistry kit (Illumina Inc., CA, USA), as per the manufacturer’s instructions. Reads were quality-filtered and passed through a demultiplexing tool to assign reads to individual wells and mapped to the TCRB and TCRA loci. TCR clonotypes were assigned using the MIXCR software prior to analysis using the visual genomics analysis studio (VGAS), an in-house program for visualising and analysing TCR data (47). Data was deconvoluted using a modified VDJfasta platform in-house at the Institute for Immunology and Infectious Diseases, Murdoch University, Australia. Large-scale contamination between plates was excluded through CDR3 overlap analysis (see **Figure S7**).

### 3’ and 5’RNA-Seq

Following the first round PCR, fragmentation and end-repair were performed using the NEBNext Ultra II FS kit (New England Biolabs, MA, USA), as per the manufacturer’s instructions. Adaptors were ligated to amplicons using the NEBNext Ultra II FS kit (New England Biolabs), as per the manufacturer’s instructions. A second round PCR was undertaken using GoTaq and linker primer 1. Following this, a third round PCR was conducted with barcoded linker primer 2. PCR products were purified and pooled for next-generation sequencing. The Kapa library quantitation kit (Kapa Biosystems Inc.) was used to quantify the libraries prior to sequencing on the Illumina MiSeq sequencer using the 600V3 kit (Illumina Inc.), as per the manufacturer’s instructions. Reads were quality-filtered and passed through a demultiplexing tool assigning reads to wells and 5’ or 3’ end. Reads were then aligned to the human reference genome GRCh38 (Ensembl rel. 92) using CLC Genomics Workbench (CLC Bio v.11, QIAGEN Bioinformatics). The sum of gene-specific 3’ and 5’ read counts were calculated using HTSeq-count and the latest Gencode annotations. Only cells with a read count >500 were included in transcriptome analyses. Furthermore, cells with less than 200 genes and more than 5% mitochondrial content were removed. Downstream analyses (normalization, PCA, differential expression and visualization) were performed in R (Seurat v.2.3.4). TSNE analyses were performed using the following parameters: theta=0, max_iter=10000 and perplexity=100. Differential expression analyses were performed using the Wilcoxon rank-sum test. P values were adjusted for multiple testing using the false discovery rate (FDR) correction. Median read counts (IQR) and averages are presented in **Table S5**. Raw sequencing data are available on the SRA database, accession number PRJNA684958.

### Cellular Indexing of Transcriptomes and Epitopes by Sequencing (CITE-Seq)

CITE-seq analysis was performed using the CITE-seq and cell hashing protocol version 2019-02-13 available on the 10x Genomics website (https://cite-seq.com/protocol/). Resting PBMCs were stained with 31 TotalSeq-C antibodies (BioLegend) at a concentration of 0.5X, as per the protocol. RNAseq and antibody sequencing was performed at the Vanderbilt University Medical Centre IMGSCT core. Demultiplexing was performed using the HTODemux function within Seurat. Antibody analysis was performed using Cell Ranger’s feature barcoding analysis pipeline (10X Genomics, CA, USA) with the resultant antibody counts normalized in the R package “Seurat” (v3.1) using centered-log-ratio normalization. RNAseq analysis was performed as described above with normalized antibody counts superimposed on the single cell RNAseq data.

### HIV-1 Sequencing

TFV sequences were inferred from the plasma of individuals during acute HIV infection using a single genome amplification (SGA) method, as described previously (48). Briefly, viral RNA was extracted from 140uL acute infected plasma by using QIAamp Viral RNA Mini Kit (Qiagen). Full-Length cDNA was generated with the SuperScript III Reverse Transcriptase (Life Technologies) and serially diluted prior to conducting PCR. All amplicons and clones were Sanger sequenced. The TFV sequences were then aligned and analyzed using Geneious Prime v2019.0.3.

For viruses persisting in plasma collected at chronic infection, HIV Gag and Nef sequences were amplified by nested PCR as previously described (41). Briefly, 4µL of sample was used in an RT-PCR with the SuperScript III One-Step RT-PCR System with Platinum Taq High Fidelity Kit (Life Technologies), as per the manufacturer’s instructions. PCR amplicons were sequenced using next-generation sequencing (as described above).

### TCR Clonality

Clonality was calculated by dividing the number of unique productive CDR3s by the total number of productive CDR3s and subtracting this value from 1. Normalising the clonality scores provides a measure of diversity that ranges from 0 to 1, with 0 and 1 representing an infinitely diverse and monoclonal population, respectively.

### Cloning of gBlocks

Full-length CDR3 regions for TCR-α and -β chains were determined from single cell TCR sequencing and custom synthesized into gBlocks (Integrated DNA Technologies, Coralville, Iowa USA) to be cloned into pSelect-GFP-Zeo vectors. The gBlock was cut with the restriction enzymes Sal-I and Nhe-I with 10x Cut smart buffer (NEB) and incubated for 2 hours at 37°C. Cut products were purified using the Qiagen PCR Purification Kit (Qiagen), as per the manufacturer’s instructions. Briefly, five volumes of buffer PB was added to the digested sample and added to the purification column. The column was washed with buffer PE and purified product eluted with 25µL of elution buffer. The insert was ligated to the similarly cut vector using 2x quick ligase buffer (NEB) and incubated for five to 15 minutes at room temperature. The ligated product was transformed into DH5alpha cells by a heat shock method. The ligation mix was added to 100µL of DH5alpha cells and incubated for 30 minutes on ice. The mix is then heat shocked at 42°C for 45 seconds then placed on ice for two minutes. Nine hundred microliters of S.O.C. Medium (ThermoScientific) was added to the tube prior to shaking for one hour at 250rpm at 37°C. Two hundred microliters of culture were plated on Agar plates (Fast-Media Zeo Agar; LB based agar medium supplemented with Zeocin; InvivoGen, CA, USA) and incubated overnight at 37°C.

Six to eight colonies were picked and cultured individually overnight in 3mL of Fast Media Zeo TB (TB-based liquid medium supplemented with Zeocin; InvivoGen) at 37°C and 250rpm. Plasmid DNA was extracted using the Wizard Plus SV Minipreps DNA Purification System kit (Promega Corporation, WI, USA), as per the manufacturer’s instructions. Briefly, culture was pelleted and resuspended in cell resuspension solution by vortex and the cells lysed with lysis buffer. Alkaline phosphate buffer was added, mixed, and incubated for five minutes at room temperature. Neutralization solution was added, with the lysate centrifuged and the supernatant put into the column. The columns were then washed twice with wash buffer and the final plasmid DNA eluted with water. Plasmid DNA was diluted to 50ng/µL and sequenced using Sanger sequencing with the primers SV40pAnR for 3’ and pSelect for 5’ to confirm the TCR sequence.

Following confirmation of insert, 1µL of a 1:10 dilution of plasmid DNA was added to 100µL DH5alpha cells and heat shocked, as above. A single colony was added to 120mL of TB-zeo medium and cultured overnight at 37°C and 250rpm. Plasmid DNA was extracted using the PureYield Plasmid Maxiprep System kit (Promega Corporation), as per the manufacturer’s instructions. Briefly, cells were pelleted and resuspended in cell resuspension solution by vortex. Cells were lysed and incubated at room temperature before the addition of the neutralization solution. The lysate was centrifuged at room temperature, with the supernatant poured into the clearing column and vacuum applied. The column was washed with endotoxin removal wash, column wash, and then dried. Once dry, plasmid DNA was eluted with water.

### Transfection of Effector Jurkat E6.1 Cells

Jurkat E6.1 T cells were cultured and passaged at least once at 0.3×10^6^cells/mL before transfection in RPMI supplemented with 10% FBS, 1% L-glutamine and 1% penicillin streptomycin. Cells were pelleted and washed twice in Opti-MEM prior to resuspension at 5×10^7^cells/mL. Jurkat cells were electroporated using Gene Pulser Xcell Electroporation System (Bio-Rad Laboratories, Inc.) using the protocol SQR wave, 230V, 25ms, 1 pulse with CE module (V, % droop) in 200µL of Opti-MEM with four plasmids: CD8alpha (5mg); TCR alpha (3mg); TCR beta (3mg); and NFAT luciferase (10mg). Electroporated cells were rested for 10 minutes prior to a 24-hour incubation in 5.8mL RPMI (no phenol red) supplemented with 10% FBS and 1% L-glutamine at 37°C and 5% CO_2_.

### TCR Reporter Assay

Specific TCR combinations were tested for their resultant induction of NFAT-driven luciferase output using a previously described protocol (23). For peptide presentation, single antigen lines for the relevant HLA allele were cultured and passaged at least once at 0.3×10^6^cells/mL before peptide pulse in RPMI supplemented with 10% FBS, 1% L-glutamine and 1% penicillin streptomycin. Cells were washed with RPMI (no phenol red) supplemented with 10% FBS and 1% L-glutamine and resuspended at 1×10^6^ cells/mL, with 50,000 cells added to each well of a U-bottom 96 well plate. Peptides at a final concentration of 30ng/µL (with serial 10-fold dilutions) were added and incubated overnight at 37°C and 5% CO_2_ in a final volume of 70µL. After 24 hours, transfected Jurkat E6.1 cells were counted and resuspended at 1×10^6^ cells/mL, with 50,000 cells being added to target cells in a final volume of 100µL. Cells were co-cultured for six hours and transferred to a white Cliniplate (ThermoScientific) prior to the addition of 100µL Bright-Glo (Promega) with an incubation of 10 minutes in the dark at room temperature. Luciferase output was measured on a DTX 880 multimode detector (Beckman Coulter) using 3000ms integration. PMA/Ionomycin was used as a positive control with four negative controls: 1) media only; 2) Jurkat only; 3) Jurkat and APCs; and 4) Jurkat and peptide. All conditions were in duplicate.

### Bioinformatics and Statistics

Graphpad Prism 8 was used for statistical analyses for **Figure 2** and **Figures S1** and **S2**. RStudio (RStudio team 2015) Version 1.1.463 for PC was used for all statistical analyses (Kruskal-Wallis Test) for **Figures 3**, **4** and **Figure S4** (49). Statistical significance threshold was set at α = 0.05 with all analyses performed on normalized gene expression counts.

Differential gene expression analyses were performed using VGAS and default parameters of the R “MAST” package were used (50). This package compares the average gene expression between two groups to identify if a gene is significantly upregulated in one group compared to the other. To account for multiple comparisons, p-values were adjusted using false discovery rate (FDR). Significant DEGs had a fold change >|2| and an FDR<0.05. Visualisation of DEGs was generated using the R “EnhancedVolcano” package (51), allowing genes to be plotted based on p-value and fold change.

The UMAP dimension reduction technique was used to visualize single cell RNA-seq clusters using the R “UMAP” package (52). The default settings with the distance metric ‘cosine correlation’ were used, as this metric was most appropriate for the dataset. UMAPs were created to reduce the data to 2-dimensions and cluster cells based on transcriptome similarity (51).

A non-parametric, unsupervised method was implemented to estimate the variation of pre-defined gene sets (Nanostring CAR-T characterization panel; https://www.nanostring.com/) using the R “GSVA” package (53). Gene set variation analysis (GSVA) takes genes associated with a specified pathway and identifies their location in a ranked list of genes (differential gene expression results are taken and ranked from highest to lowest fold change). This method identifies if genes within a gene set are enriched at one end of the ranked list or another words, enriched in one comparison, computing an enrichment score (ES). ESs were calculated using the default ‘GSVA’ method as previously defined (54). GSVA scores of single cell RNA transcriptome data were analysed and visualized using the R package “ComplexHeatmap” (55). Pearson correlation distance metrics were used for row and column hierarchical clustering. For GSVA visualisation, only significant gene sets (Kruskal-Wallis Test; p value < 0.05) between comparisons were shown. Biological pathways common between comparisons were visualised with the R package “nVennR” (56) and ggplot2.

## Data Availability Statement

The datasets presented in this study can be found in online repositories. The names of the repository/repositories and accession number(s) can be found here: https://www.ncbi.nlm.nih.gov/, PRJNA684958.

## Ethics Statement

The studies involving human participants were reviewed and approved by Vanderbilt University Medical Center and University of Alabama at Birmingham. Institutional review board (IRB) approval for sample collection was obtained prior to the commencement of the original study (Vanderbilt University Medical Center, IRB 030005; University of Alabama at Birmingham, IRB X981027004, X160125005 and X140612002). Subsequent ethics approval was obtained for the use of archived material in the repository at Vanderbilt University Medical Center (IRB 100061). All subjects signed written informed consent prior to participation.

## Author Contributions

Conceptualization: MJ, SM, SK, PG, and SG. Data curation: JC, KQ, RG, CW, LB, EA, MP, AS, LY, and SG. Formal Analysis: JC, BL, KQ, and SG. Funding acquisition: SK, PG, and SG. Software: SL and RR. Supervision: AB, SH, AC, SK, PG, and SG. Visualization: JC, BL, KQ, SL, SK, and SG. Writing – original draft: JC and SG. Writing – review & editing: JC, BL, KQ, MJ, PG, SK, and SG. All authors contributed to the article and approved the submitted version.

## Funding

The funders had no role in study design, data collection and analysis, decision to publish, or preparation of the manuscript. This work was funded by: the National Health and Medical Research Council award number APP1148284 to SG and supports BK; the University of Western Australia award number 000915/12105102 to SG; the Tennessee Center for AIDS Research award number P30 AI110527 to SK; the National Institutes of Health award numbers R01 AI112566 and AI064060 and R56 Al143482 to PG; and the University of Alabama at Birmingham award numbers R24 AI067039 and P30 AI027767 to PG.

## Conflict of Interest

The authors declare that the research was conducted in the absence of any commercial or financial relationships that could be construed as a potential conflict of interest.

## Publisher’s Note

All claims expressed in this article are solely those of the authors and do not necessarily represent those of their affiliated organizations, or those of the publisher, the editors and the reviewers. Any product that may be evaluated in this article, or claim that may be made by its manufacturer, is not guaranteed or endorsed by the publisher.

## Supplementary Material

Supplementary Table 1 IFNɣ responses to tested epitope by ICS.

Supplementary Table 2 TCR α/β combinations and overlap within subjects. ^^^Only cells containing an α and β chain were included; ^#^multiple α and β chain CDR3s were observed in a number of wells; ^*^No significant difference between NAE- and AE-specific CD8^+^ T cells (p=0.16; paired t-test); overlap is between all three conditions where relevant; where there was no clear dominant clone no percentage has been given.

Supplementary Table 3 Longitudinal sequencing of the autologous virus for subjects with multiple sampling timepoints and linked clinical data#. ^#^Additional timepoints from the sorted sample were sequenced using Sanger sequencing; - data not available; ^&^time in weeks for acute subjects and years for chronic subjects; *mixture; ^VL undetectable; ^$^specific ART regime was not available for all subjects; ART, antiretroviral therapy; subject C3 only has one timepoint; timepoint used for analyses is in bold; subjects on treatment at the sampling timepoint commenced at this time.

Supplementary Table 4 HLA class I genotypes for the loci HLA-A, -B and –C.

Supplementary Table 5 Median read count (interquartile range) and average per well by subject included for RNAseq analysis for TL10, RF10 and TY8.

Supplementary Figure 1 TCR α/β combinations for TL10- and RF10-specific CD8^+^ T cells are cross-reactive for the NAE and AE form but show no difference in TCR antigen specific reactivity to the HLA-peptide TCR complex. Flow cytometry plots for NAE- and AE-activated CD8^+^ T cells indicate similar proportions of antigen-specific CD8^+^ T cells for subject C2 for the epitope TL10 **(A)** and subject C4 for the epitope RF10 **(B)** (negative controls included). Common TCR CDR3 α/β combinations were observed in all three conditions for subjects C2 and C4 **(C)** (each color represents a single CDR3 combination). The remaining combinations unique to each condition were grouped with the number of combinations listed. **(D)** Circos plots depict the TCR α/β CDR3 combinations observed in each condition with tested CDR3 combinations colored to match those depicted in panel C (highlighted with an asterisk). The remaining TCR α/β CDR3 combinations common to all conditions are highlighted in dark grey (note, the grey is not linked to the grey of panel **C**). β chains are depicted on the bottom, with α chains depicted on the top. The width of each band correlates with the frequency of the respective α/β combination. **(E)** There was no difference in antigen specific reactivity observed in the peptide dilution series of a common TCR α/β CDR3 combination in subject C2 for the NAE and AE peptide of TL10 and similarly for subject C4 for RF10 (**F**; result for AE peptide was significantly lower than for the NAE peptide at a concentration of 0.1ng/uL; one-way ANOVA; p=0.02) using a T cell reporter assay (CDR3 combination tested is marked with an asterisks in panel **C**). The negative control is transfected Jurkat cells with antigen presenting cells minus peptide. RLU = relative light units. Note that RLU values cannot be compared across TCRs due to variability in plasmid transfection rates (see *Materials and Methods* for details).

Supplementary Figure 2 No differences in TCR CDR3 clonality between NAE- and AE-activated CD8^+^ T cell populations. **(A)** There was no difference in clonality scores for TCRs identified after stimulation with either the NAE or AE peptide for TL10, RF10 and KY11 (N = 7; p=0.20; paired t-test). **(B)** There was no difference in the clonality of resting cross-reactive (dual tetramer^+^) T cells of chronic compared to acute HIV-1 infected subjects for TL10, RF10 and TY8 (N = 7; p=0.13; unpaired t-test).

Supplementary Figure 3 T cell memory subtype composition of tetramer^+^ and activated antigen-specific T cells based on CCR7 and CD45RO expression. **(A)** Four T cell subtypes were distinguished using CCR7 and CD45RO expression by flow cytometric analysis of index-sorted cells. **(B)** T cells were predominantly of a specific memory phenotype across all conditions for a subject with Tem being the most predominant across all cells sorted. Note, Teff could not be differentiated into Tem and Temra subtypes based on the markers used. Here, only TL10-specific CD8^+^ T cells from subjects C1 and C2 are shown.

Supplementary Figure 4 Characterization of resting and activated memory CD8^+^ T cells cross-reactive to NAE and AE forms of TL10. **(A)** Box plots depict the expression of marker genes that identifies immune functionality (pro-inflammation, activation and exhaustion markers) and sex (N = 3). * p<0.05 and ^ p<0.05 Kruskal–Wallis test on rested and activated memory T cells, respectively. Note TNFRSF9 encodes the protein CD137. **(B)** Flow cytometric visualization of PD-1 surface expression (N = 3).

Supplementary Figure 5 CITE-seq analysis of un-activated cells. Protein expression **(A)** versus transcript **(B)**. The initial panel displays clustering of the cells. The most distinct lack of concordance between protein and RNA expression is observed for CD4. The remaining protein and RNA expression are similar, allowing RNA expression levels to inform protein expression.

Supplementary Figure 6 Example representation of flow gating for sorting by tetramers or activation markers (peptide stimulation) and cell subtype determination. **(A)** Gating strategy for TL10 tetramer sorting for subject 1. **(B)** Gating strategy for activation markers after TL10 NAE peptide stimulation for subject C2. Gating strategies are the same for all subjects. T cell subtype determination was the same regardless of sorting strategy and were based on CD45RO and CCR7 expression.

Supplementary Figure 7 CDR3 overlap within a subject reflecting cross-reactive NAE- and AE-specific CD8^+^ T cells. All productive CDR3s were compared between sample plates to identify possible public clonotypes and/or contamination. Overlaps were predominantly restricted to between conditions within a subject (denoted with thick black boxes), however there was evidence of a likely public clonotype for the RF10 epitope. A specific TCR alpha and beta pair was found in the chronic (C4) and acute (A1) subject that is likely to be a public clonotype. Note the alpha chain paired with other beta chains in each of the two subjects but these combinations were subject-specific. N = 10.
