# Supplementary figures and images for: Cross-Reactivity to Mutated Viral Immune Targets Can Influence CD8+ T Cell Functionality: An Alternative Viral Adaptation Strategy
Source: Front Immunol. 2021 Oct 26;12:746986. doi: 10.3389/fimmu.2021.746986 (PMC8577586; doi:10.3389/fimmu.2021.746986)

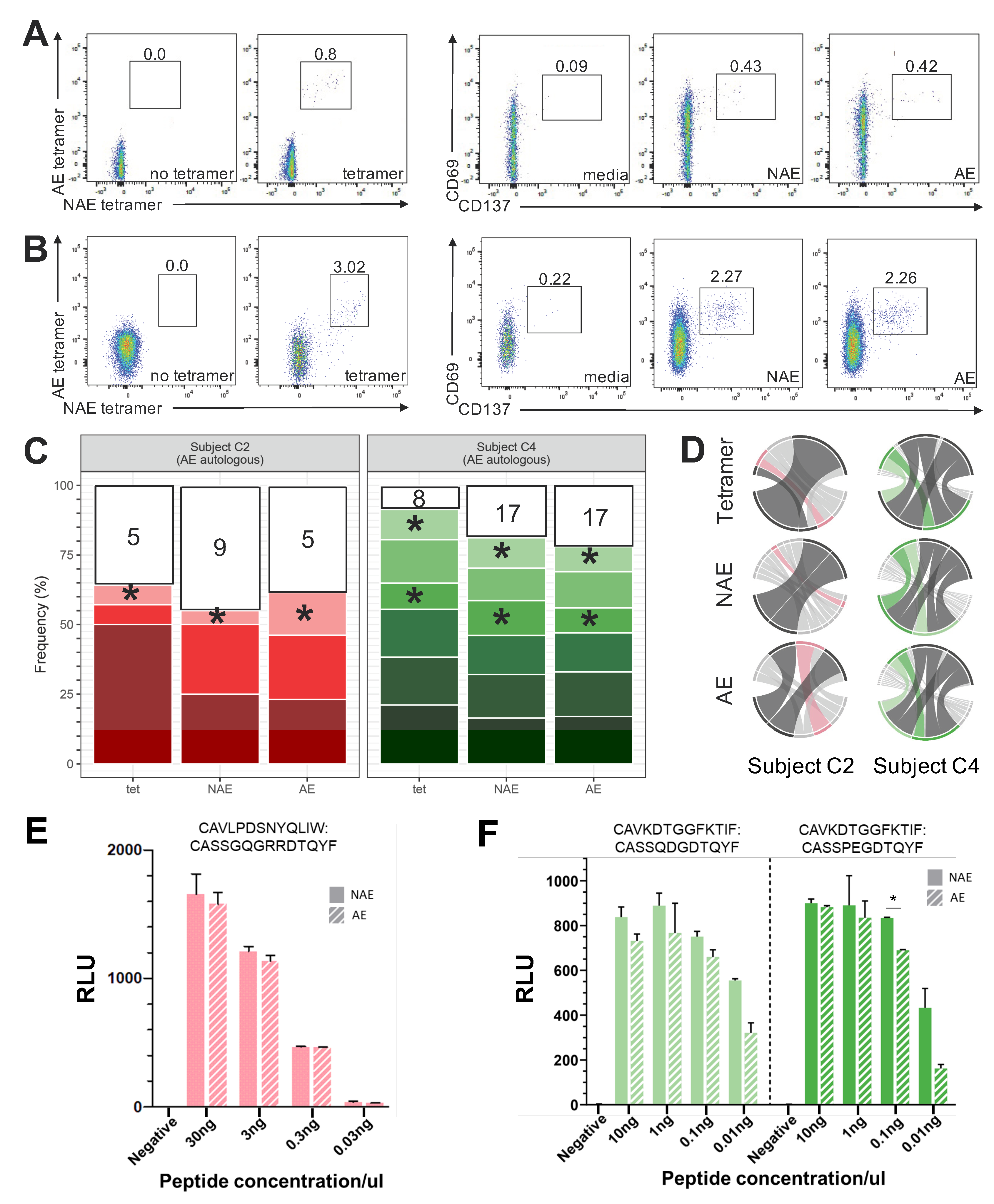

Supplement: Supplementary Figure 1 — TCR α/β combinations for TL10- and RF10-specific CD8+ T cells are cross-reactive for the NAE and AE form but show no difference in TCR antigen specific reactivity to the HLA-peptide TCR complex. Flow cytometry plots for NAE- and AE-activated CD8+ T cells indicate similar proportions of antigen-specific CD8+ T cells for subject C2 for the epitope TL10 (A) and subject C4 for the epitope RF10 (B) (negative controls included). Common TCR CDR3 α/β combinations were observed in all three conditions for subjects C2 and C4 (C) (each color represents a single CDR3 combination). The remaining combinations unique to each condition were grouped with the number of combinations listed. (D) Circos plots depict the TCR α/β CDR3 combinations observed in each condition with tested CDR3 combinations colored to match those depicted in panel C (highlighted with an asterisk). The remaining TCR α/β CDR3 combinations common to all conditions are highlighted in dark grey (note, the grey is not linked to the grey of panel C). β chains are depicted on the bottom, with α chains depicted on the top. The width of each band correlates with the frequency of the respective α/β combination. (E) There was no difference in antigen specific reactivity observed in the peptide dilution series of a common TCR α/β CDR3 combination in subject C2 for the NAE and AE peptide of TL10 and similarly for subject C4 for RF10 (F; result for AE peptide was significantly lower than for the NAE peptide at a concentration of 0.1ng/uL; one-way ANOVA; p=0.02) using a T cell reporter assay (CDR3 combination tested is marked with an asterisks in panel C). The negative control is transfected Jurkat cells with antigen presenting cells minus peptide. RLU = relative light units. Note that RLU values cannot be compared across TCRs due to variability in plasmid transfection rates (see Materials and Methods for details). [file Image_1.tiff]

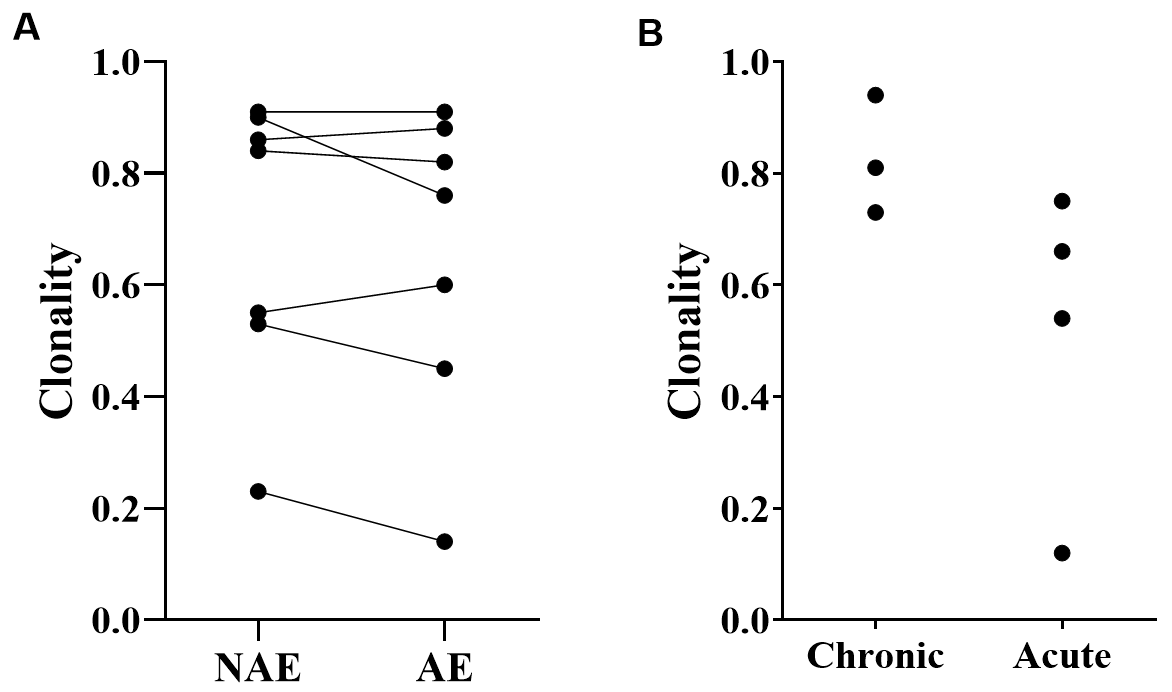

Supplement: Supplementary Figure 2 — No differences in TCR CDR3 clonality between NAE- and AE-activated CD8+ T cell populations. (A) There was no difference in clonality scores for TCRs identified after stimulation with either the NAE or AE peptide for TL10, RF10 and KY11 (N = 7; p=0.20; paired t-test). (B) There was no difference in the clonality of resting cross-reactive (dual tetramer+) T cells of chronic compared to acute HIV-1 infected subjects for TL10, RF10 and TY8 (N = 7; p=0.13; unpaired t-test). [file Image_2.tif]

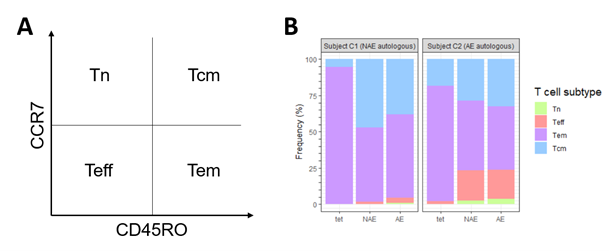

Supplement: Supplementary Figure 3 — T cell memory subtype composition of tetramer+ and activated antigen-specific T cells based on CCR7 and CD45RO expression. (A) Four T cell subtypes were distinguished using CCR7 and CD45RO expression by flow cytometric analysis of index-sorted cells. (B) T cells were predominantly of a specific memory phenotype across all conditions for a subject with Tem being the most predominant across all cells sorted. Note, Teff could not be differentiated into Tem and Temra subtypes based on the markers used. Here, only TL10-specific CD8+ T cells from subjects C1 and C2 are shown. [file Image_3.tif]

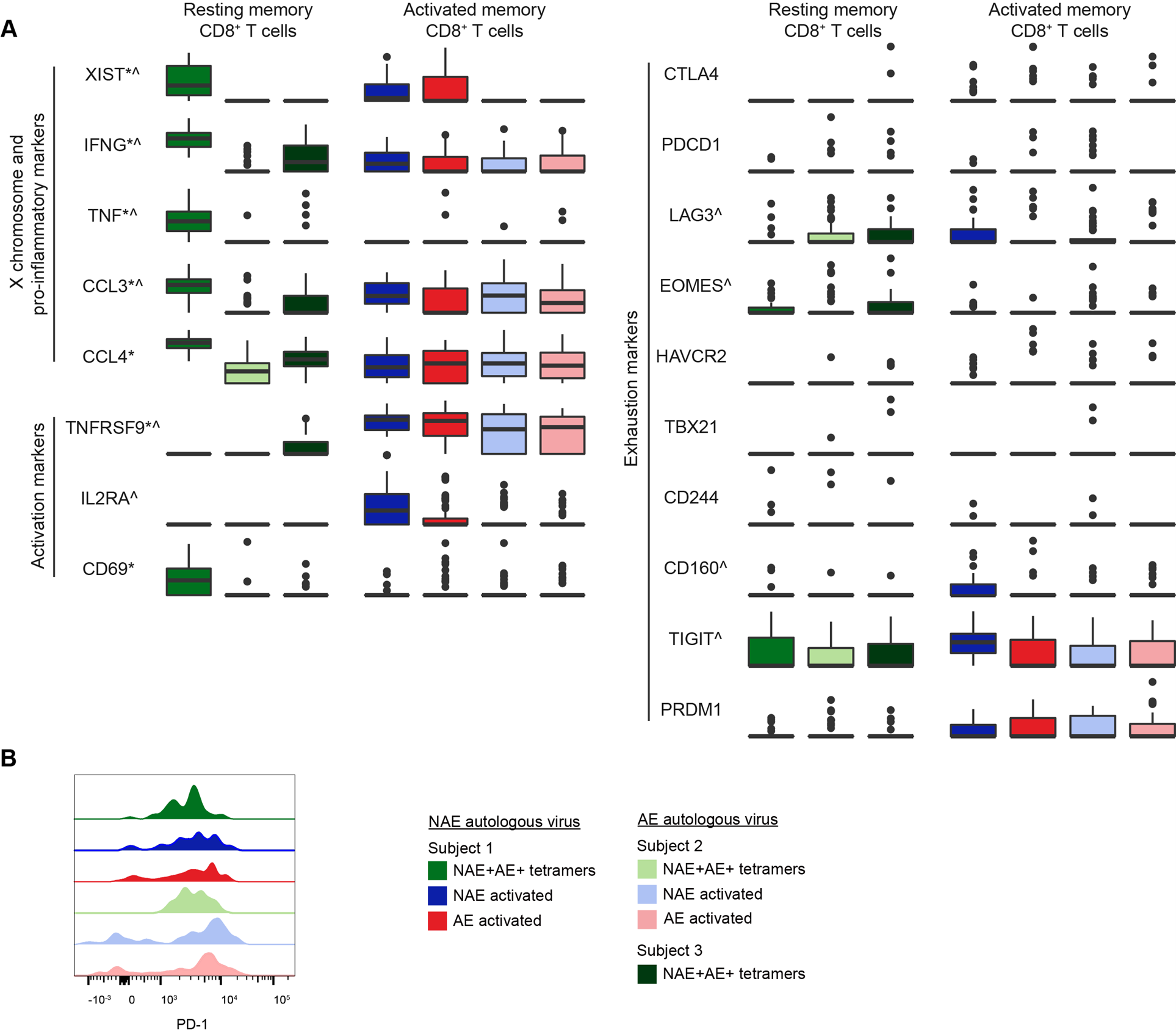

Supplement: Supplementary Figure 4 — Characterization of resting and activated memory CD8+ T cells cross-reactive to NAE and AE forms of TL10. (A) Box plots depict the expression of marker genes that identifies immune functionality (pro-inflammation, activation and exhaustion markers) and sex (N = 3). * p<0.05 and ^ p<0.05 Kruskal–Wallis test on rested and activated memory T cells, respectively. Note TNFRSF9 encodes the protein CD137. (B) Flow cytometric visualization of PD-1 surface expression (N = 3). [file Image_4.tif]

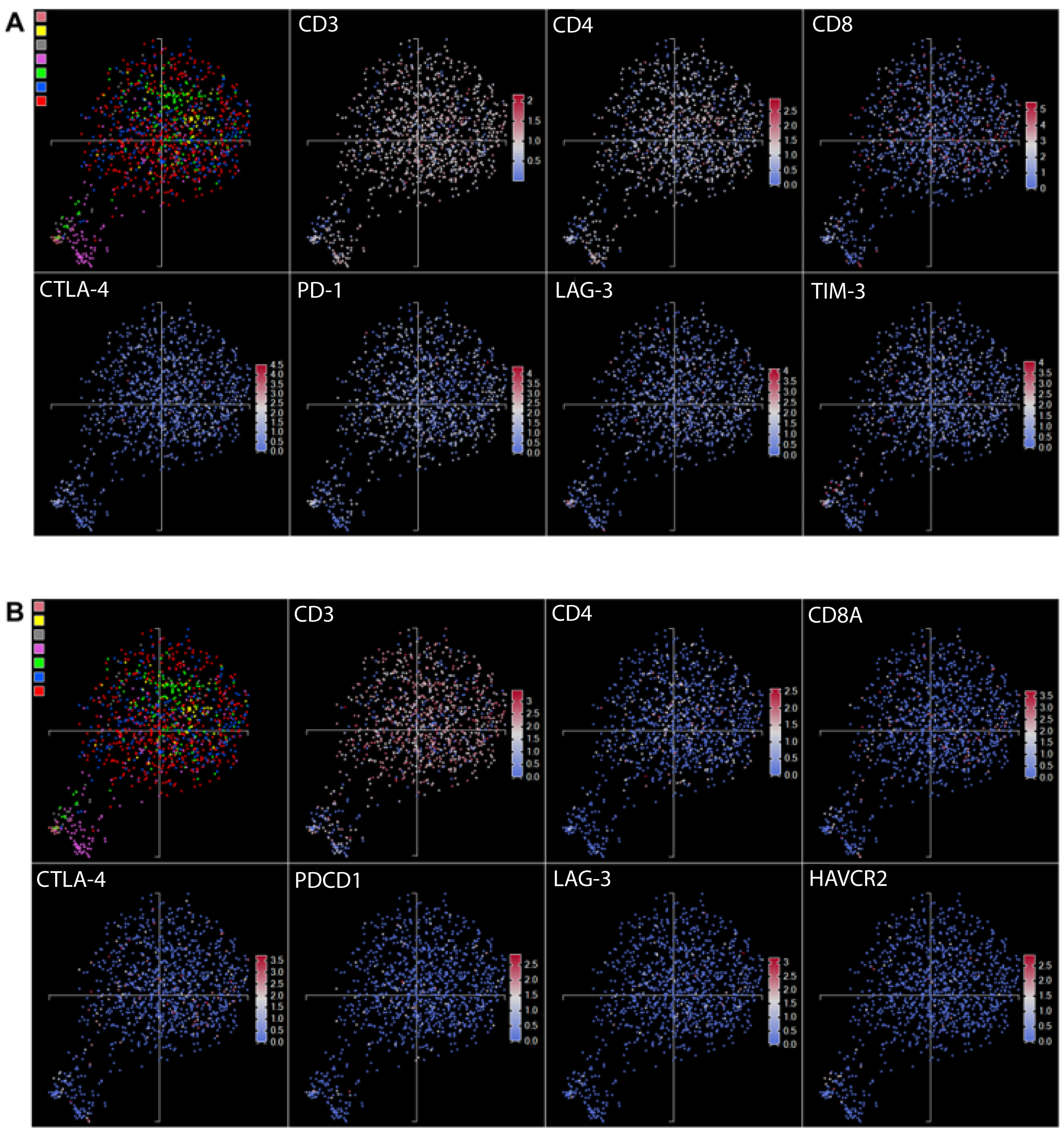

Supplement: Supplementary Figure 5 — CITE-seq analysis of un-activated cells. Protein expression (A) versus transcript (B). The initial panel displays clustering of the cells. The most distinct lack of concordance between protein and RNA expression is observed for CD4. The remaining protein and RNA expression are similar, allowing RNA expression levels to inform protein expression. [file Image_5.tif]

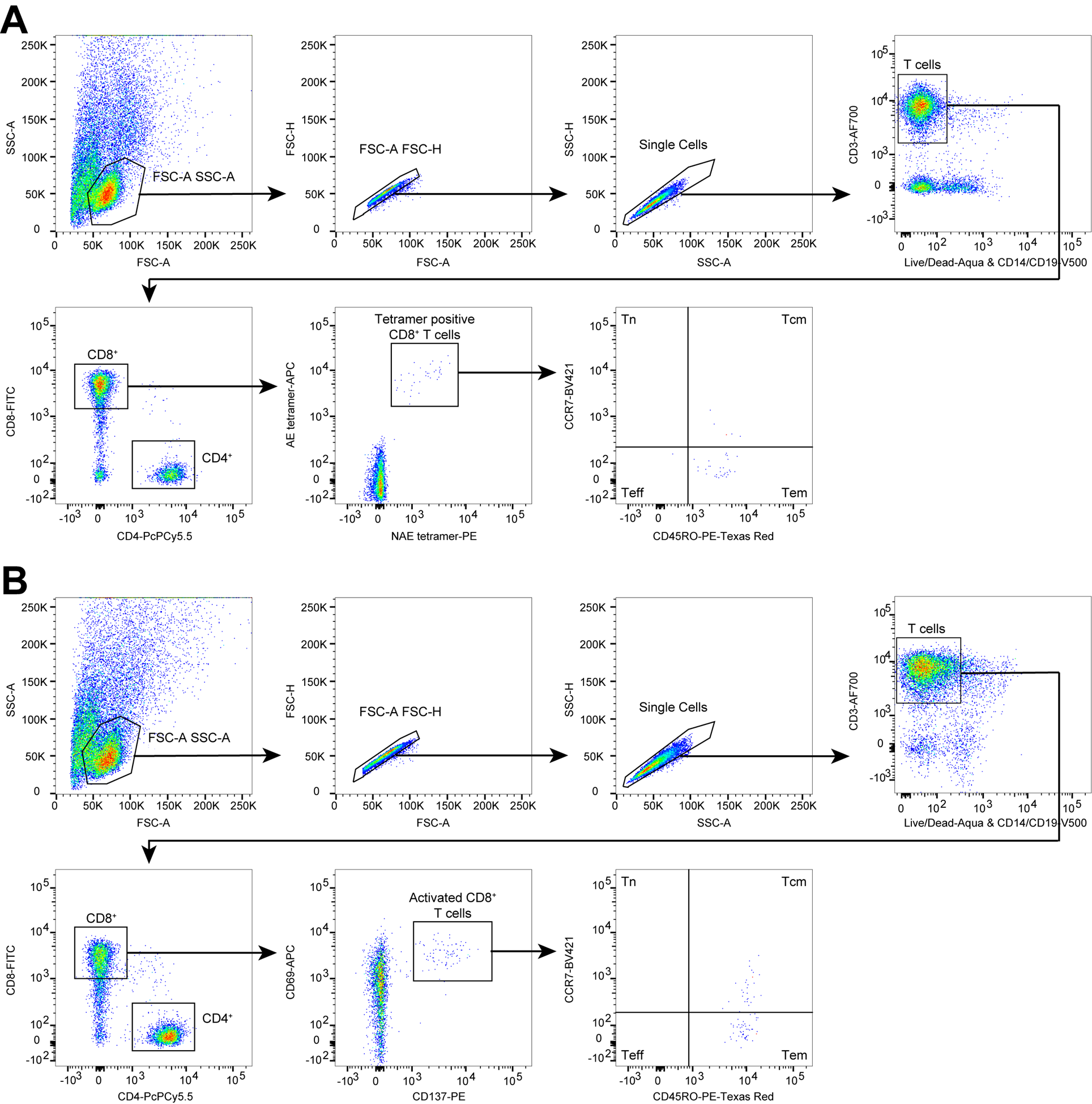

Supplement: Supplementary Figure 6 — Example representation of flow gating for sorting by tetramers or activation markers (peptide stimulation) and cell subtype determination. (A) Gating strategy for TL10 tetramer sorting for subject 1. (B) Gating strategy for activation markers after TL10 NAE peptide stimulation for subject C2. Gating strategies are the same for all subjects. T cell subtype determination was the same regardless of sorting strategy and were based on CD45RO and CCR7 expression. [file Image_6.tif]

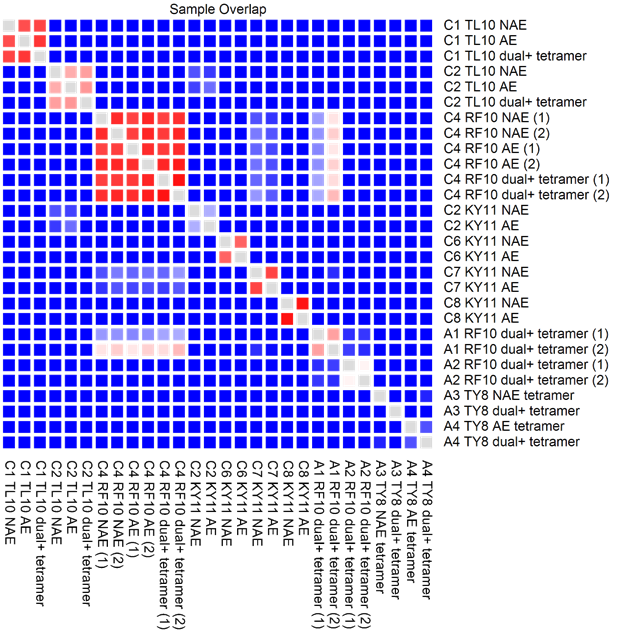

Supplement: Supplementary Figure 7 — CDR3 overlap within a subject reflecting cross-reactive NAE- and AE-specific CD8+ T cells. All productive CDR3s were compared between sample plates to identify possible public clonotypes and/or contamination. Overlaps were predominantly restricted to between conditions within a subject (denoted with thick black boxes), however there was evidence of a likely public clonotype for the RF10 epitope. A specific TCR alpha and beta pair was found in the chronic (C4) and acute (A1) subject that is likely to be a public clonotype. Note the alpha chain paired with other beta chains in each of the two subjects but these combinations were subject-specific. N = 10. [file Image_7.tif]
